# Rock fragmentation indexes reflecting rock mass quality based on real-time data of TBM tunnelling

**DOI:** 10.1038/s41598-023-37306-7

**Published:** 2023-06-27

**Authors:** Xu Li, Lei-jie Wu, Yu-jie Wang, Jin-hui Li

**Affiliations:** 1 Key Laboratory of Urban Underground Engineering of the Ministry of Education, Beijing Jiaotong University, Beijing, 100044 China; 2 Department of Geotechnical Engineering, China Institute of Water Resources and Hydropower Research, Beijing, 100038 China; 3 School of Civil and Environmental Engineering, Harbin Institute of Technology (Shenzhen), Shenzhen, 518055 China

**Keywords:** Engineering, Civil engineering

## Abstract

Perception of rock condition (RC) is a challenge in tunnel boring machine (TBM) construction due to lack of space and time to observe and detect RC. To overcome this problem, this study aims to extract a new rock fragmentation index (RFI) that can reflect RC from real-time rock fragmentation data of the TBM. First, a comprehensive review of existing rock fragmentation models is conducted, which leads to some candidate RFIs that can reflect RC. Next, these candidate RFIs are investigated using data from 12,237 samples from a well-monitored tunnel boring process of the TBM in a 20,198 m tunnel. Further, a new RFI system is recommended as the parameter involving the optimal models. Finally, a preliminary study of the relationship between these RFIs and RC is carried out, and it is shown that these RFIs can reflect RC to a large extent. In the TBM boring process, these RFIs can be extracted from real-time TBM fragmentation data and used to predict the RC in the field. Therefore, the challenge of RC perception is solved with this new RFI system. The new RFI system offers significant potential for the real-time rock classification, prediction of the surrounding rock collapse potential, and selection of control parameters or support measures during TBM construction. This will be the key to improving TBM construction performance.

## Introduction

With the rapid development of water conservation, highway, and railway infrastructure in China, the number of long tunnels under construction is increasing^1^. More than 200 long water diversion or transport tunnels will be constructed in China over the next decade. For example, the Kangding–Linzhi section of the Sichuan-Tibet railway, which is currently under construction, includes six long tunnels with lengths over 30 km in length. Developing an efficient and safe construction method for long tunnels is important for the smooth and rapid completion of these projects.

If the surrounding rock conditions (RC) of the tunnel are good, the maximum monthly advance of full-face rock tunnel boring machine (TBM) construction can exceed 1800 m, which is more than five times the monthly advance of the traditional drilling and blasting methods. Because of this advantage, TBM is considered the preferred construction method for long tunnels in rock strata^2,^^3^.

However, the construction efficiency and safety of TBMs are very sensitive to changes in the surrounding RC in the field. When adverse geological conditions^4–6^ are encountered, serious safety accidents such as machine jamming^7,8^ or rock bursts^9–11^ may frequently occur. If hard rock is encountered or the construction control parameters are not adjusted in time according to the RC, a massive disc cutter consumption^12,^^13^ will occur. Therefore, the perception of the RC is key to improving the construction efficiency and safety of TBMs.

**Figure 1:**
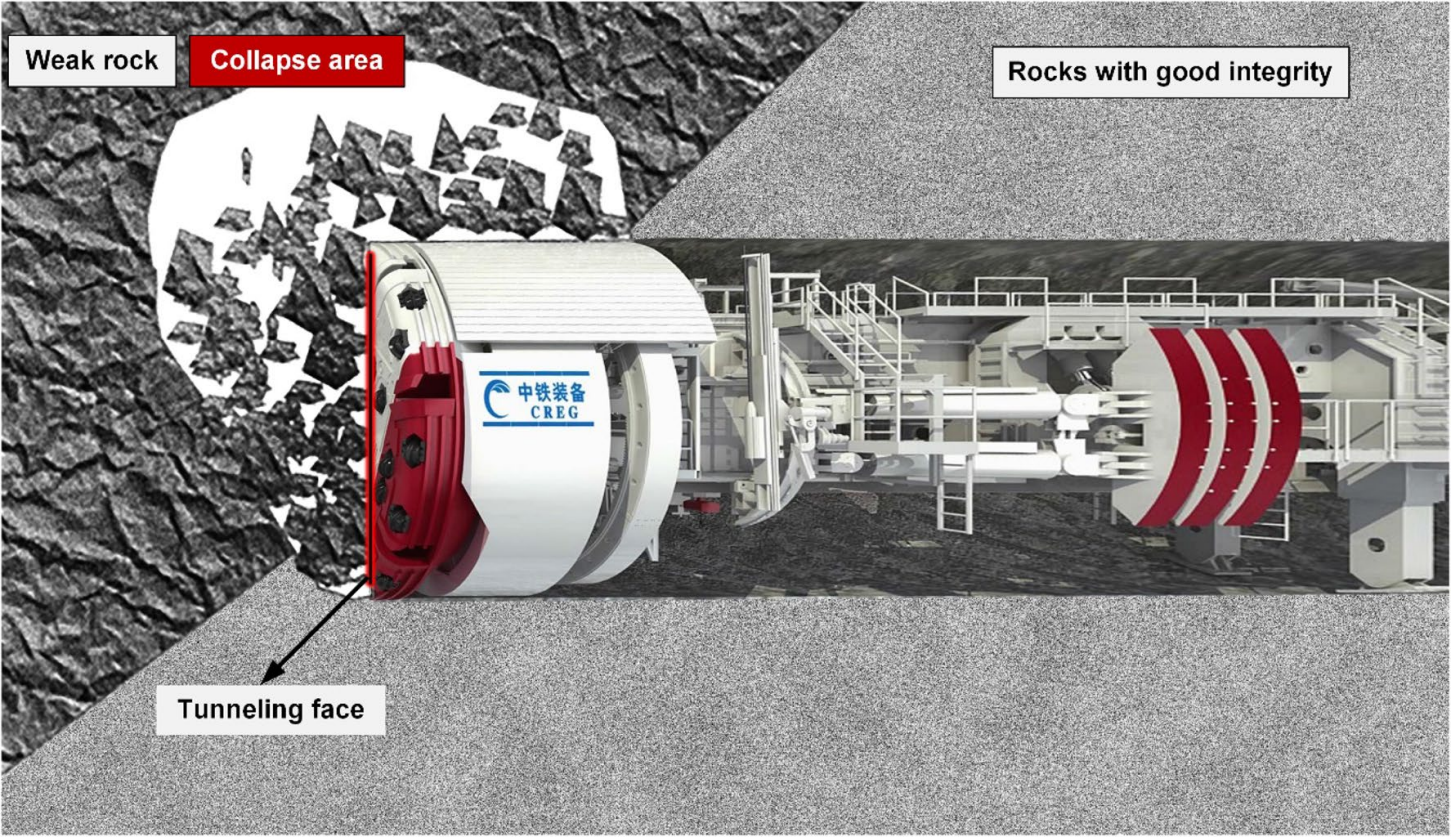
Perception of rock conditions is a challenge in TBM construction.

At present, the perception of RC remains a challenge in TBM construction. As shown in Fig. 1, TBM construction is carried out rapidly in a closed and confined space. There is lack of space and time to observe and detect RC. Traditional RC perception methods, such as rock surface observation, field testing, and advanced geological prediction technology are not applicable to TBM construction.

To overcome this challenge, a potential perception method for RC is to judge RC in real time based on the rock fragmentation data of the TBM construction. When the relationship between rock fragmentation data and RC, i.e., the TBM rock fragmentation model, is established. RC perception based on rock fragmentation data can be realised, which can be used to guide TBM construction, improve construction efficiency, take necessary engineering measures in time, and avoid accidents such as machine jamming.

Many efforts have been made to study the relationship between RC and rock fragmentation data or to establish a TBM rock fragmentation model, including numerical simulations^14^, theoretical studies^15,16^, and laboratory and field tests^17–19^. Several rock fragmentation models (including the CSM and NTNU models) focus more on the predicting of penetration rate (PR)^20^ and cutter life^21,^^22^ than on the prediction of torque and thrust. In this study, only the predicting of torque (or thrust) prediction is considered, and PR prediction is not discussed.

Rock fragmentation models that address torque (or thrust) can be divided into the following categories.

**Figure 2:**
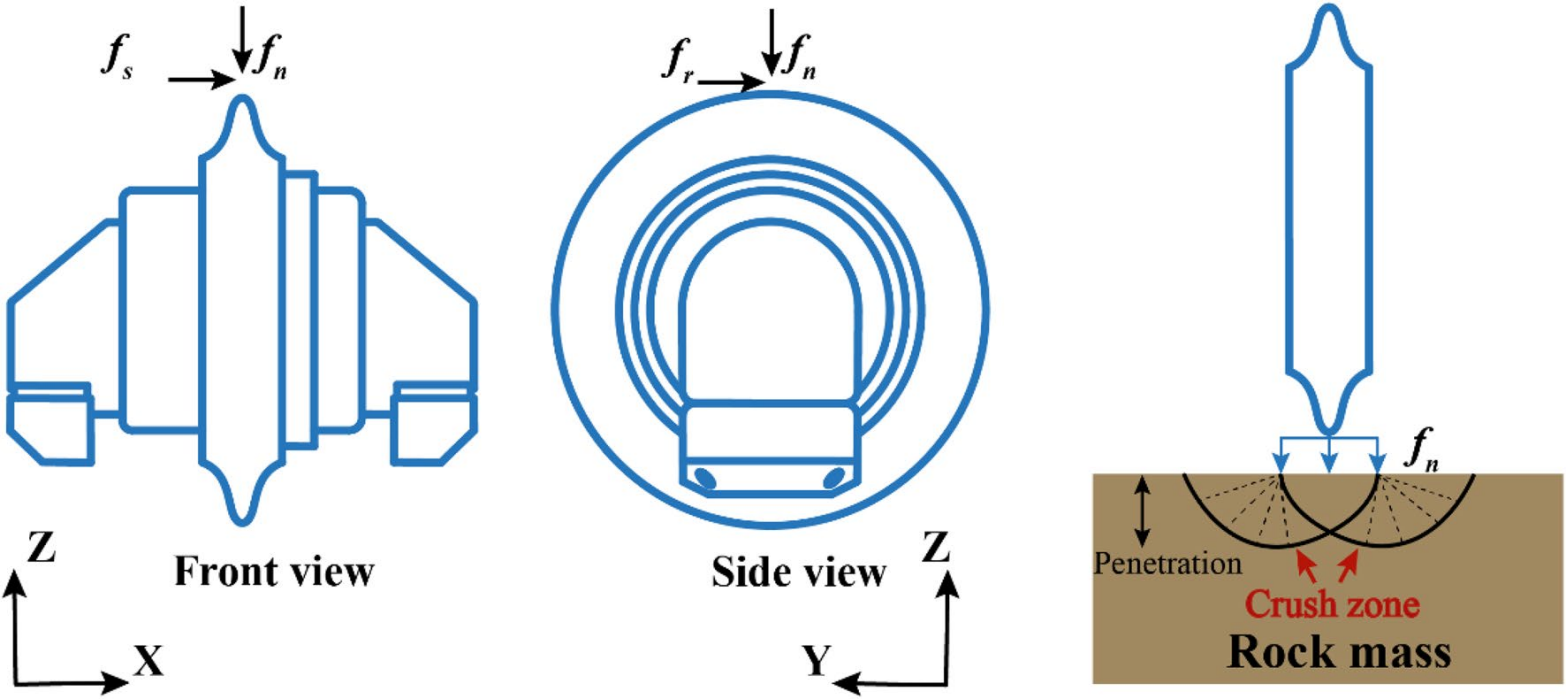
Forces at work on disc cutter.

*Individual cutter models.* The most used mechanical models are the individual cutter models. Such models are proposed based on the mechanical analysis of the rock fragmentation process on a complete rock. Rock fragmentation varies depending on penetration $p$, which is defined as the cut depth for each revolution of the cutterhead. Hence, many individual cutter models propose formulas to characterize the relationship between forces and penetration, as shown in Appendix 1. In these models, ${f}_{r}$, ${f}_{n}$, and ${f}_{s}$ are the rolling force, normal force, and side force acting on the disc cutter, respectively (Fig. 2). In these formulas, the parameters depend on the rock properties^16,23,24^ and other factors.

*Empirical holistic models.* In addition to the individual cutter model, some empirical holistic models have addressed the relationship between the forces applied on the cutterhead and penetration. Such empirical models consider the entire TBM cutterhead as an integrated system. The representative empirical models for the cutterhead are listed in Appendix 2. Such models use regression to establish the relationship between total forces and penetration. In such models, the parameters depend on the rock properties^25–29^ and some other factors.

These models significantly improve our understanding of rock fragmentation mechanisms^30^. These studies have demonstrated that the TBM can be regarded as a large torsional shear testing machine for rock and rock fragmentation data dependent on RC. However, the relationship between RC and rock fragmentation data is complex and includes factors, such as the influence of construction control parameters, disc cutter shape, and disc cutter arrangement,in addition, the requirement of rock properties increases the difficulty in information gathering and limits the model’s application. Although the current research results and formulas have partially solved this problem and are applicable under some special conditions, a widely applicable rock fragmentation model has not been found.

Due to the complex nature of rock fragmentation, over 10 different models have been proposed to address this problem. These models vary in detail and may even contain contradictory or conflicting information. Even for experts in the field, it is still unclear which model is the most superior and universal. Thus, the development of a universal rock fragmentation model remains a challenging task in TBM construction.

In our opinion, there is another way to properly use these models, that is the model parameters can be regarded as rock fragmentation indexes (RFIs) and determined by model fitting using real-time TBM fragmentation data. In addition, RFIs can be used to reflect the RC.

Studies on RFIs have been well summarised and discussed by Hassanpour et al.^31^, Hamidi et al.^32^, and Farrokh et al.^33^. Among these RFIs, the field penetration index (*FPI*) has been well discussed^33^. Hamilton and Dollinger^34^ first introduced the *FPI* and defined it as the total thrust over the penetration. Later, several relationships between the RC (such as the volumetric joint count and rock mass rating value) and *FPI* were obtained by regression^31,35,36^. Later, Delisioetal.^37^ adopted *FPI* to examine the surrounding RC and TBM penetration behaviour.

In short, we believe that extracting RFIs from real-time TBM fragmentation data and using them to predict RC is the key to improving TBM construction performance. A single index of *FPI* cannot fully represent the complex RC. All parameters involved in the rock fragmentation models are potential RFIs that can reflect RC. Thus, a full comparison and verification of real-time TBM fragmentation data is required.

To propose a widely applicable rock fragmentation model, this study aims to extract TBM RFIs that can reflect RC from real-time rock fragmentation data and verify the universality of the proposed RFI through big data of TBM construction. The “Rock fragmentation data” is a collective term for the relevant sensor parameters collected by the TBM when breaking rock masses.

The remainder of this study is structured as follows. (1) In “TBM construction data in YinSong diversion project (YSP)” section , big data of TBM construction is introduced; (2) in “Rock fragmentation model from a macro energy consumption perspective” section , new energy consumption relations for the rock fragmentation process of TBM are deduced from a macro perspective; (3) in “Model adaptation test based on TBM construction data in YSP” section , the big data of TBM construction is used to verify the universality of these rock fragmentation models and lead to a new RFI system; (4) finally, in “Benefits of the newly proposed TBM rock fragmentation index system” section , the benefits of the new RFI system are discussed.

## TBM construction data in YinSong diversion project (YSP)

With the rapid advancement of the TBM sensor technology^38^, data on the TBM boring process can be collected. With the support of China's National Basic Research Program (973 Program), the TBM boring process was well monitored. The high-quality data obtained in the YSP opens a data-driven possibility for establishing relationships between the TBM rock fragmentation data and RC. This section introduces the rock fragmentation data of the YSP,subsequently, these data were used to evaluate the rock fragmentation models.

### Geological conditions and TBM used in YSP

#### Engineering survey of the TBM3 LOT in YSP

YSP is located the Jilin Province, China. It is a water diversion project from the Fengman Reservoir to the centre of Jilin Province. The main channel of this project is 263.45 km. The maximum tunnel diameter is 7.9 m, and the tunnel bottom slope is approximately 1/4300. The average overburden depth of the tunnel is between 50 and 100 m, with a maximum overburden depth of 260 m. From the Fengman reservoir to the Yinma River, three river valleys divide the 72.1 km line into three mountain sections of nearly equal length. Each mountain section was excavated by a separating TBM^39,^^40^.

**Figure 3:**
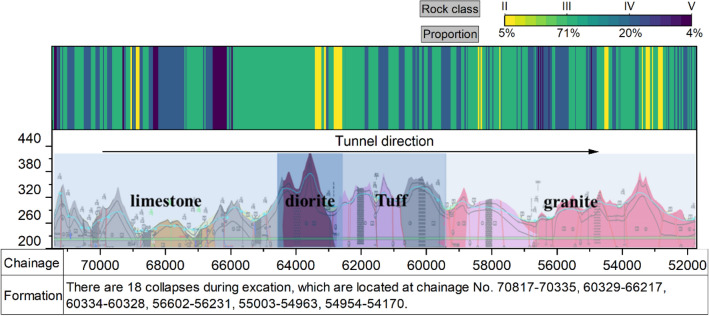
Geological stratigraphic profile along the TBM3 LOT.

In this study, the data of TBM3 LOT is used. In the excavation of TBM3 LOT, four primary rock categories (Table 1) are revealed: granite (8766 m), limestone (4781 m), tuff sandstone (3448 m), and diorite (2096 m). According to the Chinese rock classification system (The National Standards Compilation Group of People’s Republic of China, 2014), the rocks of the TBM3 LOT are mainly categorised into classes II, III, IV, and V. The distributions of rock classes and their proportions are shown in Fig. 3. The field-measured physical and mechanical indexes of the rock are listed and explained in Appendix 3.

**Table 1:** Type of surrounding rock along the TBM3 LOT in YSP.

Chainage No. (m)	Length (m)	Type of surrounding rock
50,180–58,946	8766	Granite
58,946–62,394	3448	Tuff sandstone
48,900–50,180, 62,394–63,210	2096	Diorite
66,350–71,131	4781	Limestone, mudstone, sandstone, etc

Like the RMR system^41^, the Chinese rock classification system classifies rocks according to their strength, joints, water conditions, and field stress conditions. In the Chinese rock classification system, a high rock class value indicates poor rock quality. For example, rock class I refers to a rock mass with an RMR value of 100–80 and denotes a complete and fresh rock,rock class V refers to a rock mass with an RMR value in the range of 20–0 and denotes completely decomposed rock^41^. Liu et al.^1^ discussed the differences between different rock classification systems.

The RCs of the YSP are plotted in Fig. 3 and the physical and mechanical properties of various rock classes in the YSP are listed in Table 2. Because of the many surrounding rocks along the tunnel belonging to class IV or class V rock masses, 18 collapse zones occurred during the TBM boring process.

**Table 2:** Physical and mechanical properties of different rock classes in YSP.

Rock class	${k}_{0}$	$f$	$UCS$	${\sigma }_{t}$	${f}^{\mathrm{^{\prime}}}$	${c}^{\mathrm{^{\prime}}}$	${E}_{50}$	$\mu$	${V}_{P}$
	MPa/cm	–	MPa	MPa	–	MPa	GPa	–	10^3^ m/s
^*1^II_a_	[50, 80]	[7, 8]	[80, 130]	[5, 8]	[1.3, 1.4]	[1.8, 2.0]	[15, 20]	[0.22, 0.25]	> 4.5
^*2^II_b_	[40, 50]	[6, 7]	[60, 80]	[4, 6]	[1.2, 1.3]	[1.7, 1.8]	[10, 15]	[0.20, 0.25]	[4, 4.5]
^*3^III_a_	[30, 50]	[4, 7]	[60, 80]	[4, 5]	[1.1, 1.2]	[1.3, 1.5]	[8, 10]	[0.26, 0.28]	[3, 4.5]
^*4^III_b_	[20, 30]	[3, 5]	[40, 60]	[2, 4]	[0.8, 1.0]	[0.7, 1.0]	[5, 8]	[0.26, 0.30]	[3, 4]
^*5^IV	[5, 10]	[2, 3]	[10, 30]	[0.5, 1]	[0.6, 0.7]	[0.3, 0.5]	[2, 4]	0.3	[1, 2.5]
^*6^V	< 5	[0.5, 1]	< 5	< 0.3	[0.3, 0.4]	[0.05, 0.1]	[0.2, 2]	0.35	< 1

${k}_{0}$, $f$, $UCS$, ${\sigma }_{t}$, ${f}^{\mathrm{^{\prime}}}$, ${c}^{\mathrm{^{\prime}}}$, ${E}_{50}$, $\mu$, and ${V}_{P}$ denote the coefficients of the rock resistance, firmness coefficient, uniaxial compressive strength, tensile strength, friction coefficient, cohesion, deformation modulus, Poisson’s ratio, and P-wave velocity of the rock mass, respectively.

*1 class II_a_ includes granite and diorite; *2 class II_b_ includes limestone; *3 class III_a_ includes granite, diorite, and albite porphyry; *4 class III_b_ includes limestone, tuff, glutenite, and tuffaceous sandstone; *5 class IV includes limestone and tuff; *6 class V includes tuff and sandstone.

#### Equipment used in TBM3 LOT

**Figure 4:**
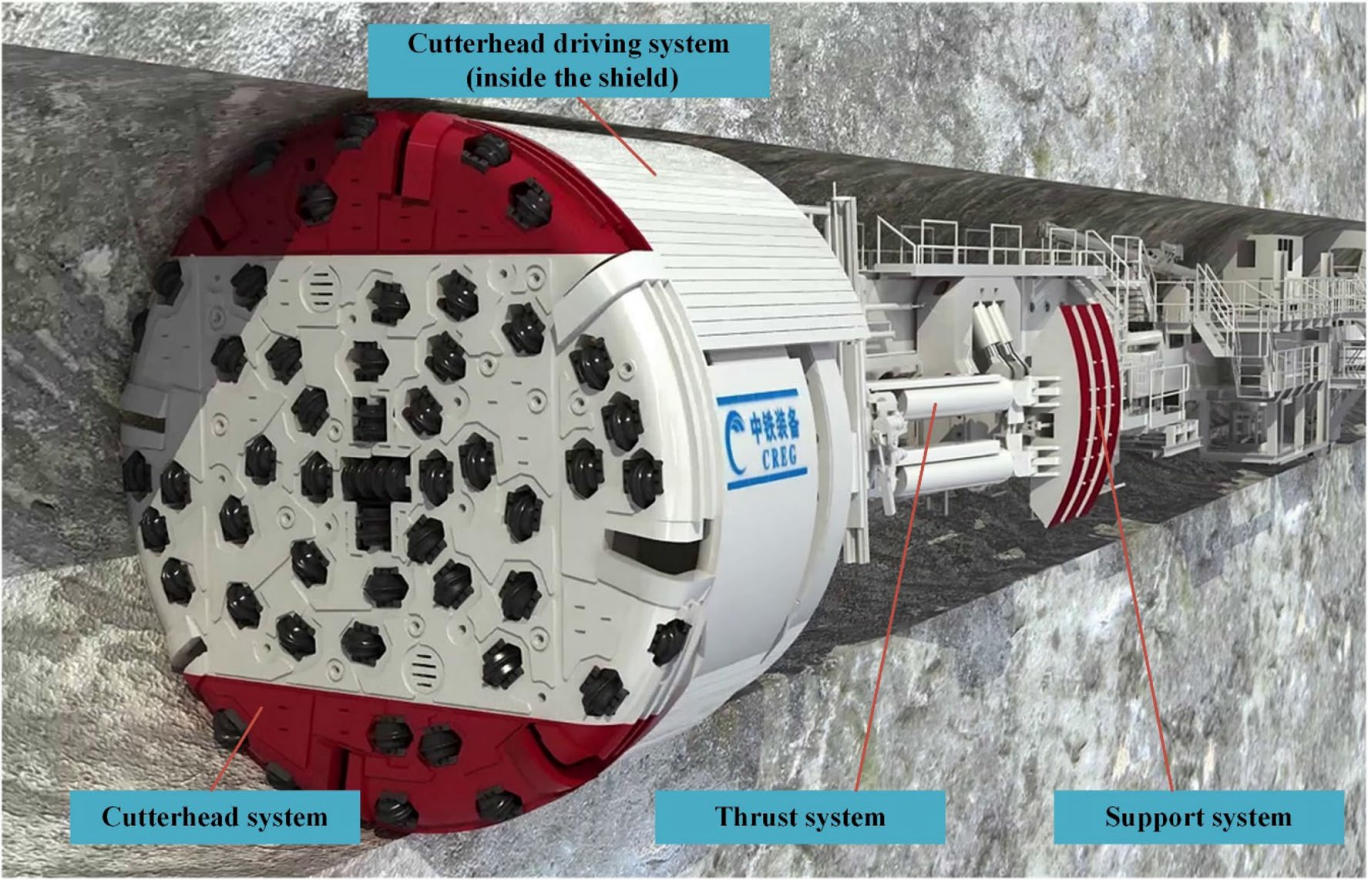
TBM equipment used in the TBM3 LOT.

The TBM3 LOT was equipped with an open TBM manufactured by the China Railway Engineering Equipment Group Co., Ltd. (CREG). Figure 4 depicts a 3-dimensional schematic diagram of the equipment. Five subsystems comprised the machine: cutterhead, cutterhead driving system, thrust system, support system, and slagging system (which is used to transport the rock pieces in front of the cutterhead out of the tunnel through a belt conveyor and is not shown in Fig. 4). The main specification of the TBM used in YSP are listed in Table 3.

**Table 3:** Main specification of equipment in TBM3 LOT.

Parameters	Value
Shield type	Open type
Cutterhead diameters	ɸ7930 mm
Number of cutters	8 pieces of 17 in., 48 pieces of 19 in
Nominal disc cutter spacing	89 mm
Thrust cylinder stroke^a^	1800 mm
Maximum thrust of cutterhead^b^	23,260 kN
Normal torque of cutterhead	8410 kN m
Instantaneous torque of cutterhead	12,615 kN m
Maximum cutterhead rotation speed	7.6 (r/min)
Maximum mucking capacity of belt conveyor	755 m^3^/h

^a^In a tunnelling cycle, the advance of the TBM cannot exceed the length of the thrust cylinder stroke, that is, 1800 mm.

^b^In the normal boring process, the TBM cannot exceed the maximum thrust of the cutterhead, normal torque of the cutterhead, maximum cutterhead rotation speed, and maximum mucking capacity of the belt conveyor.

### Rock fragmentation data in a tunnelling cycle

In the YSP, the boring process of the TBM was well monitored. In this study, only the monitored rock fragmentation data were used to establish the RFIs that reflect the RC. Let us first examine the key rock fragmentation data of the TBM.

#### Definition of a tunnelling cycle

Tunnelling cycle is defined as the standard step in the TBM boring process. The actual TBM boring process is performed step-by-step because of the limited stroke of the TBM thrust cylinder and the requirement for timely surrounding rock support.

The boring distance, that is, the TBM advance, may vary with the RC at each step (tunnelling cycle). If the rock is strong and the boring process is smooth, the advance can use the maximum stroke of the thrust cylinder, which is 1.8 m (Table 3). If the rock is weak and broken, it must be supported in time to prevent the surrounding rock from collapsing. In this case, the boring distance in a tunnelling cycle is suitably reduced. For example, in class V rock, the advance in one step is frequently reduced to around 0.5 m. Additionally, if an emergency occurs during the boring process, such as mechanical failure, the TBM operators will immediately stop the boring process, thereby resulting in a tunnelling cycle advance of less than 1.8 m.

An interval (up to 1 h) was set between a few continuous tunnelling cycles. During pause, the equipment steps back a short distance to allow the engineer to perform internal equipment checks. Moreover, if the condition of the rock is poor, a rock support system is used.

In most cases, the RC remains unchanged within a tunnelling cycle. Hence, the data from a tunnelling cycle can be analysed.

#### Key data in the TBM tunnelling process

Thrust and torque, provided by the thrust and cutterhead systems, respectively, are the major initiatives to penetrate and break the rock mass during the rock fragmentation process. TBM rock fragmentation can be divided into two stages: first, the disc cutters penetrate the rock, which results in interior cracks in the rock mass and broken fragments at the tunnel face; second, the cracks between adjacent cutters extend and join each other, and finally, large chips are formed between the two cutters^21^.

In the rock fragmentation mechanism, four independent variables are closely related to the TBM boring process (Table 4): (1) *F*, the total thrust; (2)* T*, cutterhead torque; (3)* n*, TBM cutterhead rotation speed; and (4) *v*, TBM penetration rate.

**Table 4:** Four essential data in rock fragmentation of TBM.

Name	Symbol	Unit	Type	Remarks
Cutterhead rotation speed (RPM)	$n$	r/min	Operating parameters	It is set by the TBM operator and will not change significantly during the boring process
Penetration rate (PR)	$v$	mm/min		It is set by the TBM operator and fluctuates around the set value due to the vibration of the cutterhead
Cutterhead torque	*T*	kN m	Response parameters	It is calculated using the cutterhead system’s real-time motor power and RPM
Total thrust	*F*	kN		It is calculated using the pressure and area of the thrust system’s pushing cylinder

The rock fragmentation process of the TBM cutterhead can be adopted either by the penetration control mode or force control mode^42^. The TBM employed in TBM3 LOT adopted the penetration control mode, wherein the rotation speed and penetration rate were adjusted in real time or set by the TBM operator. The torque and thrust were monitored in real time.

#### Typical data in a tunnelling cycle

**Figure 5:**
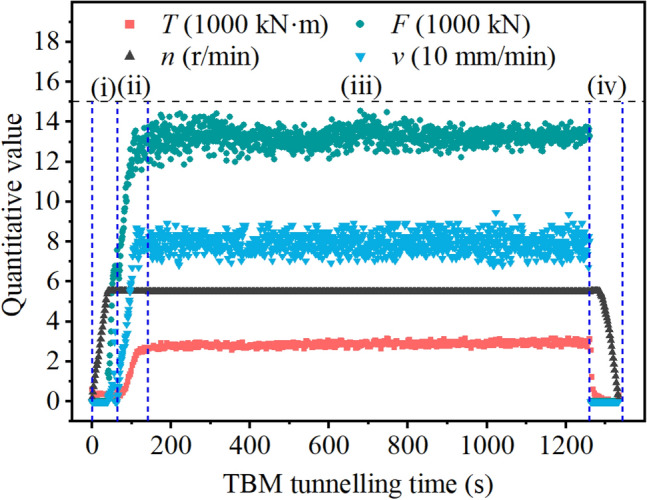
Rock fragmentation data in a tunnelling cycle.

Figure 5 illustrates the key data recorded by the TBM during a tunnelling cycle. For a tunnelling cycle, the rock fragmentation data could be divided into the following four phases^43,^^44^.

i. Free-running phase. Herein, the TBM cutterhead was not in contact with the tunnelling face; therefore, the *T* and *F* values were low.
ii. Loading phase. Herein, the TBM cutterhead advanced and gradually contact the tunnelling face. The penetration rate $v$ was higher before contact, dropped to a lower value after contact, and gradually increased to the operator’s intended value. Simultaneously, the cutterhead torque and total thrust increased progressively.
iii. Stable boring phase. Herein, the penetration process was expected to be stabilised, and the fluctuations of $v$, $T$, $F$ were expected to be small.
iv. End the boring phase. At the end of the penetration process, all parameter values decreased and plateaued at low values.

#### Importance of the loading phase

According to earlier research, $v$ is not the best parameter for rock fragmentation. Penetration has been widely used by researchers^23,^^25,^^34,^^26^ to characterise rock fragmentation behaviour. Penetration is the cutting depth of the disc cutters for each turn of the cutterhead and is defined as

1 $$p=v/n.$$

As shown in Fig. 5, in the loading phase, *n* is constant and only $v$ changes, $p$ gradually increased, and the TBM rock breaking data has a relatively wide range of values. Therefore, the loading phase can be regarded as a continuous torsional shear test of TBM equipment under different penetration values^10^.

For the same penetration $p$, the harder or more complete the rock, the greater the torque and thrust required in the rock fragmentation process. Consequently, the data of the loading phase depends on both $p$ and RC. Essentially, the data of the loading phase contain knowledge of the RC. Therefore, the data of the loading phase are the most important and valuable for TBM penetration. It is feasible to extract the knowledge of RC through the data of the loading phase, and this method can overcome the significant challenge of no space and time to observe and detect RC in the rapid TBM boring process.

### Rock fragmentation data of TBM3 LOT

#### Statistical analysis of tunnelling cycle characteristics

The boring process in the TBM3 LOT was well monitored. In the TBM3 LOT, the total length of the TBM construction section was 20,198 m, and the construction period was approximately 3.5 years. Li et al.^45^ and Jingetal.^30,^^22^ provided thorough descriptions of the data.

**Figure 6:**
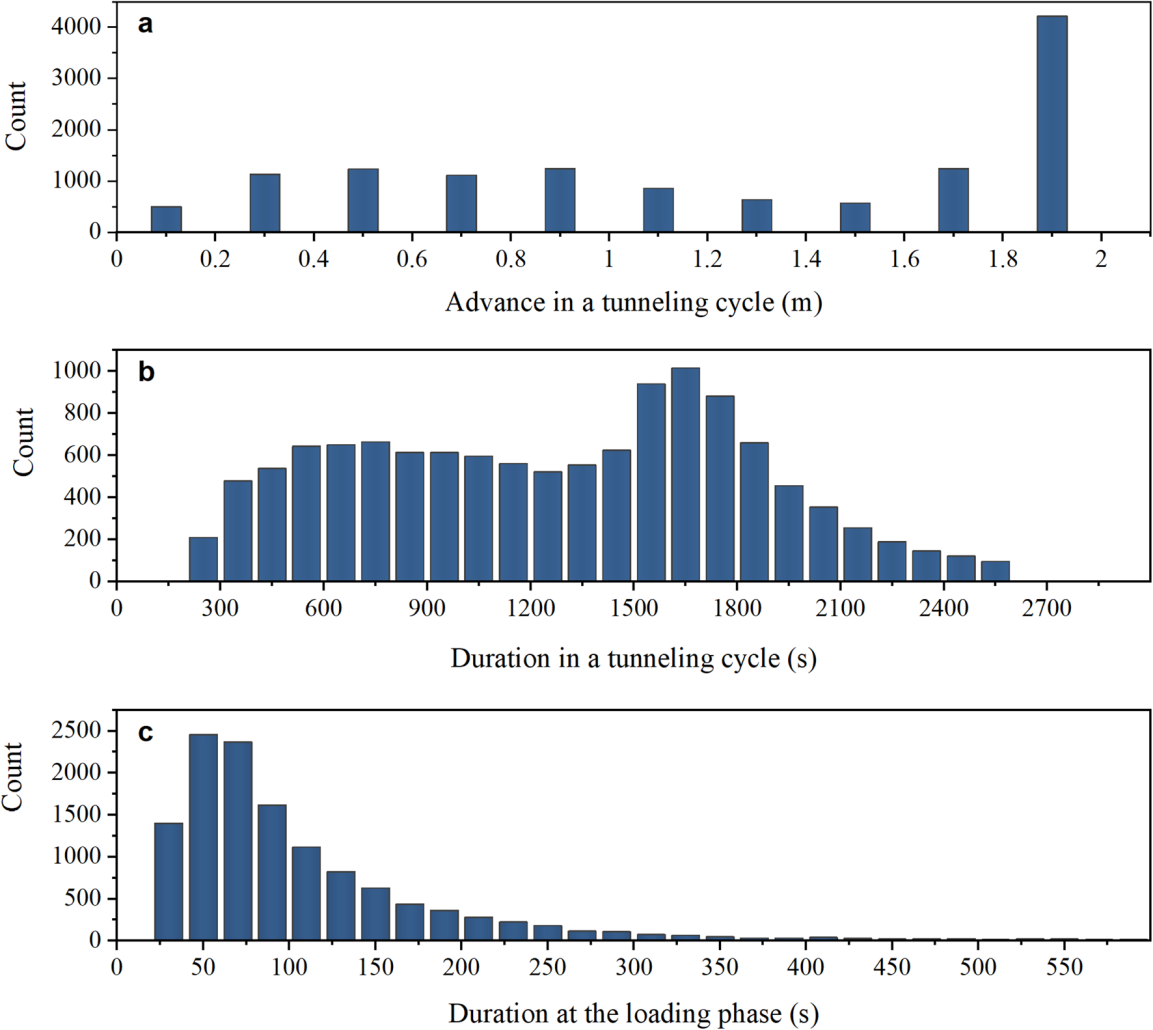
Statistical results of advance and duration in 12,237 tunnelling cycles. (**a**) advance, (**b**) duration of tunnelling cycle, and (**c**) duration of loading phase.

In this study, all the boring data from the 20,198 m tunnel excavated by TBM were recorded and analysed. First, 12,237 tunnelling cycles were discovered, and the advance and boring durations were calculated, as shown in Fig. 6.

The mean value of the advance, duration in a tunnelling cycle, and duration at the loading phase were 1.2 m, 1390 s, and 150 s, respectively. With the difference in the TBM operators’ habits and surrounding RCs, the duration of the loading phase was approximately 1–5 min.

The penetrating advance with lower values was concentrated primarily in areas with poor surrounding rocks (rock class IV or class V). In such a case, the surrounding rock should be supported in time and the TBM advance is relatively limited.

#### Examples of tunnelling data

**Figure 7:**
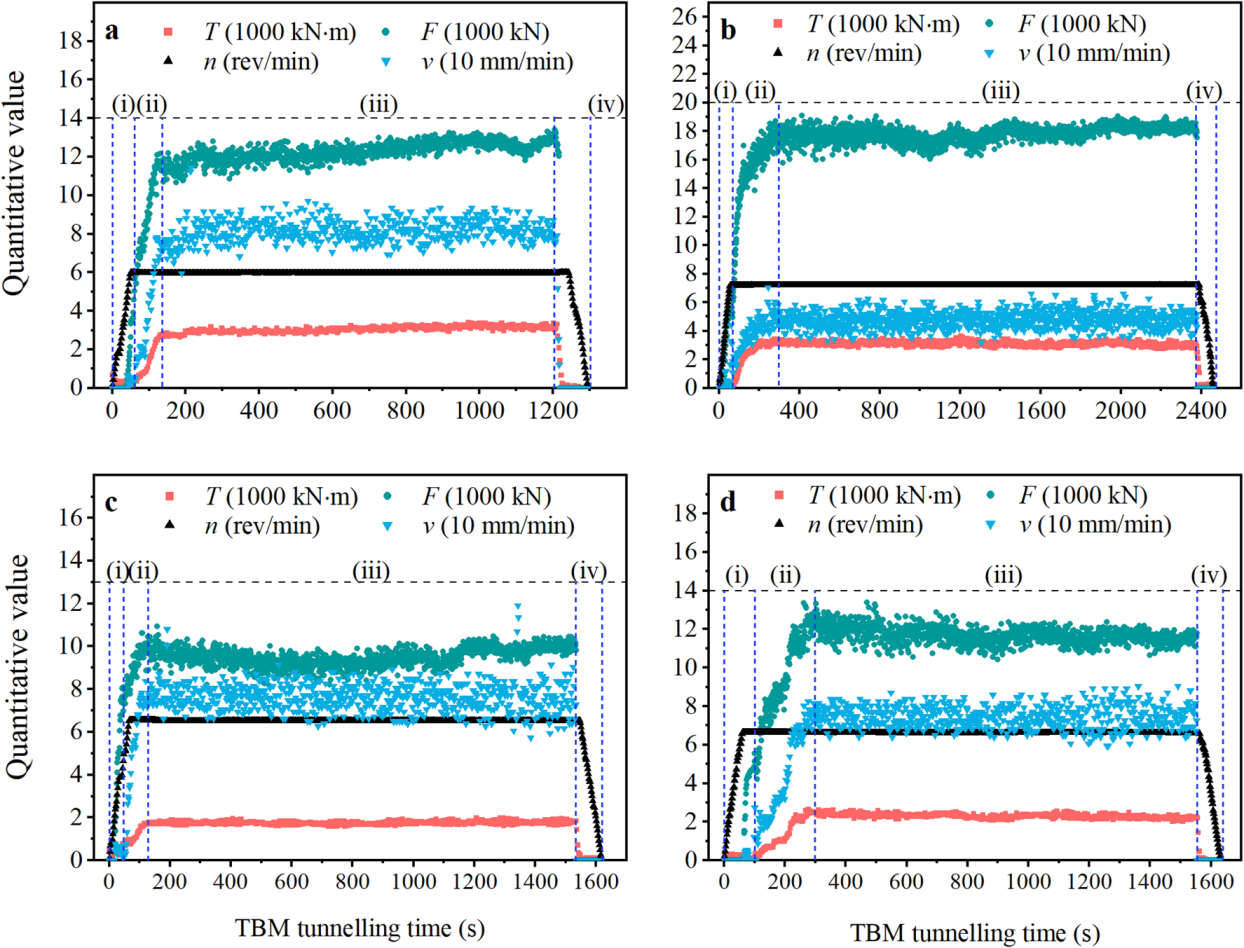
Rock fragmentation data of normal tunnelling cycles in various rock. (**a**) chainage No. 71046 (rock class III, limestone), (**b**) chainage No. 62649 (rock class II, diorite), (**c**) chainage No. 50760 (rock class III, granite) and (**d**) chainage No. 60512 (rock class IV, tuff sandstone).

The boring process is smooth (or normal) in most tunnelling cycles^46^, with distinct free running, loading, stable boring, and ending of boring phases. Records of numerous smooth boring processes, for example, are presented in Fig. 7a–d. Referring to Fig. 7, the data for various rock types and classes reflect the same characteristics as those in Fig. 5.

## Rock fragmentation model from a macro energy consumption perspective

Existing rock fragmentation models are based on mechanical analyses or empirical regression. In this section, the rock fragmentation relationship was analysed from the third perspective, that of macro energy consumption.

### Overview

According to Appendix 1, if the rock properties are given, a general form for the forces acting on a single disc cutter can be written as

2.1 $${f}_{r}={c}_{1}{p}^{{m}_{1}},$$

2.2 $${f}_{n}={c}_{2}{p}^{{m}_{2}},$$

where ${c}_{1}$ and ${c}_{2}$ are two constants that depend on the rock properties and disc cutter characteristics in a single penetrating advance; and ${m}_{1}$ and ${m}_{2}$ are two parameters that depend on the rock-fragmentation process.

Rostami^14^ provided approximate connections between the cutterhead torque and cutting forces acting on the disc cutters as well as the total thrust, as:

3.1 $$T\approx {\sum }_{k=1}^{N}{r}_{k}{f}_{r}^{k}\approx {c}_{3}N\overline{{f }_{r},}$$

3.2 $$F={\sum }_{k=1}^{N}{f}_{n}^{k}\approx N\overline{{f }_{n}},$$

where $k$ is the serial number of disc cutters; $N$ is the total number of disc cutters; ${f}_{n}^{\mathrm{k}}$ and ${f}_{r}^{\mathrm{k}}$ are the normal and cutting forces acting on the kth disc cutter, respectively; $\overline{{f }_{n}}$ and $\overline{{f }_{r}}$ are the average normal and rolling forces acting on cutters, respectively; ${r}_{k}$ is the distance between the centre of the kth disc cutter and the centre of the cutterhead (Fig. 4); and ${c}_{3}$ is a constant that varies depending on the arrangement of disc cutters and approximately equals 0.3 D.

A formula for the cutterhead torque and total thrust can be found in Appendix 2 by combining Eqs. 2.1–3.2.

4.1 $$T=\mathrm{a}{p}^{{m}_{1}},$$

4.2 $$F=\mathrm{b}{p}^{{m}_{2}},$$

where a and b are constants that depend on the rock properties in one penetrating advance and the disc cutter characteristics, respectively. There are several interpretations of the ${m}_{1}$ value in Eq. 4.1, including 1.5 in Fukuiand Okubo^47^, 2 in Roxborough and Phillips^23^, roughly 2 in the CSM model^25^, and 1 in Sanio^24^. Similarly, the ${m}_{2}$ value in Eq. 4.2 varies depending on the model, for example, 1 in Fukuiand Okubo^47^, 0.5 in Sanio^24^, and 0.5–1.5 in the CSM model^25^.

### Energy consumption relations

#### Total energy consumption relationship

The energy consumption of a cutterhead system during rock fragmentation is divided into two parts: the energy cost of rock fragmentation and the heat dissipation of friction. Because the friction torque caused by the friction between the shield and rock surface is small compared to the total torque in the rock fragmentation process, the friction heat generation between the shield and rock surface can be ignored. Therefore, the following relationship is valid.

5.1 $${W}_{T}={W}_{breakage}+{W}_{friction},$$

5.2 $${W}_{T}=T\times \omega =2\pi *T*n/60$$

5.3 $${W}_{breakage}=EvA/60,$$

5.4 $${W}_{friction}={\sum }_{\mathrm{k}=1}^{\mathrm{N}}{d}_{k}{\omega \times \xi f}_{n}^{\mathrm{k}}=2\pi /60n\xi {{\sum }_{\mathrm{k}=1}^{\mathrm{N}}{d}_{k}{f}_{n}^{\mathrm{k}}\approx 2\pi /60n\xi \mathrm{c}}_{3}N\overline{{f }_{n}}=2\pi /60n\xi {\mathrm{c}}_{3}F,$$

where ${W}_{T}$, ${W}_{breakage}$, ${W}_{friction}$ represent the work done in 1 s by cutterhead torque, rock fragmentation, and friction heat generation, respectively; $\omega$ is the radian of the rotation for the cutterhead in 1 min; *A* is the area of the tunnel face; *E* is the energy required for rock fragmentation per unit volume; $\xi$ is the coefficient of rock friction; ${\mathrm{c}}_{3}$ is a constant that depends on the arrangement of disc cutters, as reported in Eq. 3.1; and $\overline{{f }_{n}}$ is the average normal force, as shown in Eq. 3.2.

Rearranging Eqs. 5.1–5.4, the following relation can be obtained

6 $$T={I}_{c}p+{I}_{f}F,$$

where $p$ is the penetration, that is, the forward distance of the cutterhead for each rotation, and equals $v/n$; ${I}_{c}$ equals $EA/2\pi$; and ${I}_{f}$ equals $\xi {\mathrm{c}}_{3}$. If the rock properties and penetrating conditions in a tunnelling cycle are assumed to be similar, $E$ and $\xi$ are constants. For the same TBM, $A,$ and ${\mathrm{c}}_{3}$ are costants. Hence, ${I}_{c}$ and ${I}_{f}$ are constants within the same tunnelling cycle. Moreover, ${I}_{c}$ is expected to depend on rock cohesion and can be referred to as the rock cohesion index, whereas ${I}_{f}$ is expected to be dependent on the coefficient of rock friction $\xi$ and can be referred to as the rock friction index.

Using Eq. 6, one proposition of the energy-consumption relationship can be concluded as follows:

Proposition 1 (energy relationship): Cutterhead torque has a bilinear relationship with total thrust and penetration.

#### Torque penetration relation

Proposition 1 is flawed in that ${I}_{c}$ and ${I}_{f}$ are negatively correlated. Such a negative correlation will cause ${I}_{c}$ and ${I}_{f}$ to lose uniqueness, and in some cases, result in illogical values (e.g., negative values). A single-parameter relationship is more appealing for avoiding this drawback.

To accomplish this purpose, this study proposed the application of torque to rock fragmentation and friction heat generation. The relationships can be obtained as:

7.1 $${W}_{friction}={\lambda W}_{breakage,}$$

7.2 $${W}_{T}=T\times \omega =T\times n\times 2\pi /60=\left(1+\lambda \right){W}_{breakage}=\left(1+\lambda \right)EvA/60,$$

Equation 7.2 may be rewritten as:

7.3 $$T/p=\left(1+\lambda \right)*EA/2\pi ,$$

A torque penetration index (*TPI*) can be established using Eq. 7.3 is as:

7.4 $$TPI=T/p=\left(1+\lambda \right)*\frac{EA}{2\pi }=const.$$

Another proposition of energy consumption relation may be concluded using Eq. 7.4, as follows.

Proposition 2 (TPI relationship): Cutterhead torque penetration index is a constant.

Throughout Eq. 7.4, two strong assumptions were made: (1) $\lambda$ is a constant in the TBM penetration process, and (2) *E* is a constant in a tunnelling cycle. These two assumptions are likely to be violated if the rock properties or boring conditions vary throughout a tunnelling cycle. When Eq. 7.4 is applied to the TBM construction data, the single unknown parameter, that is, *TPI* can be directly calculated using the known *T* and* p*. Consequently, only one unknown parameter relationship exists.

Referring to Eq. 3.1, *T* is linear to the average normal and cutting forces acting on the cutter $\overline{{f }_{r}}$. Thus, another physical meaning of Eq. 7.4 that $\overline{{f }_{r}}$ is linear to the penetration $p$.

#### Energy consumption ratio

Torque and thrust energy are consumed during the penetration process. A new assumption may be introduced: the ratio of torque work to thrust work is a constant related to RC. This assumption can be expressed as:

8.1 $$WR={W}_{T}/{W}_{F}=const,$$

where ${W}_{F}$ is the work done by thrust and may be represented as,

8.2 $${W}_{F}=\left(F*v\right)/\left(6\times {10}^{4}\right),$$

Equations 8.2 and 7.2, can be substituted into Eq. 8.1 to produce the following relationship.

8.3 $$WR={W}_{T}/{W}_{F}=2\pi \times {10}^{3}\left(T*n\right)/\left(F*v\right)=const.$$

The proposition can be defined using Eq. 8.3 is as follows.

Proposition 3 (Work ratio relationship): The ratio of work done by cutterhead torque to the work done by total thrust is constant. According to Eq. 8.3, this is equivalent to the ratio of *T* to (F*p) is constant. Additionally, this proposition describes the relationship between cutterhead torque, total thrust, and penetration. Herein, supposing that Eq. 8.3 is adapted to the TBM construction data, *WR* is the only unknown parameter that can be calculated directly from the known data, including *T, F, n,* and* v*. Like proposition 2, Proposition 3 is a single-unknown parameter relationship.

### Preliminary verification of three proposed propositions

#### Verification by the data in YSP

**Figure 8:**
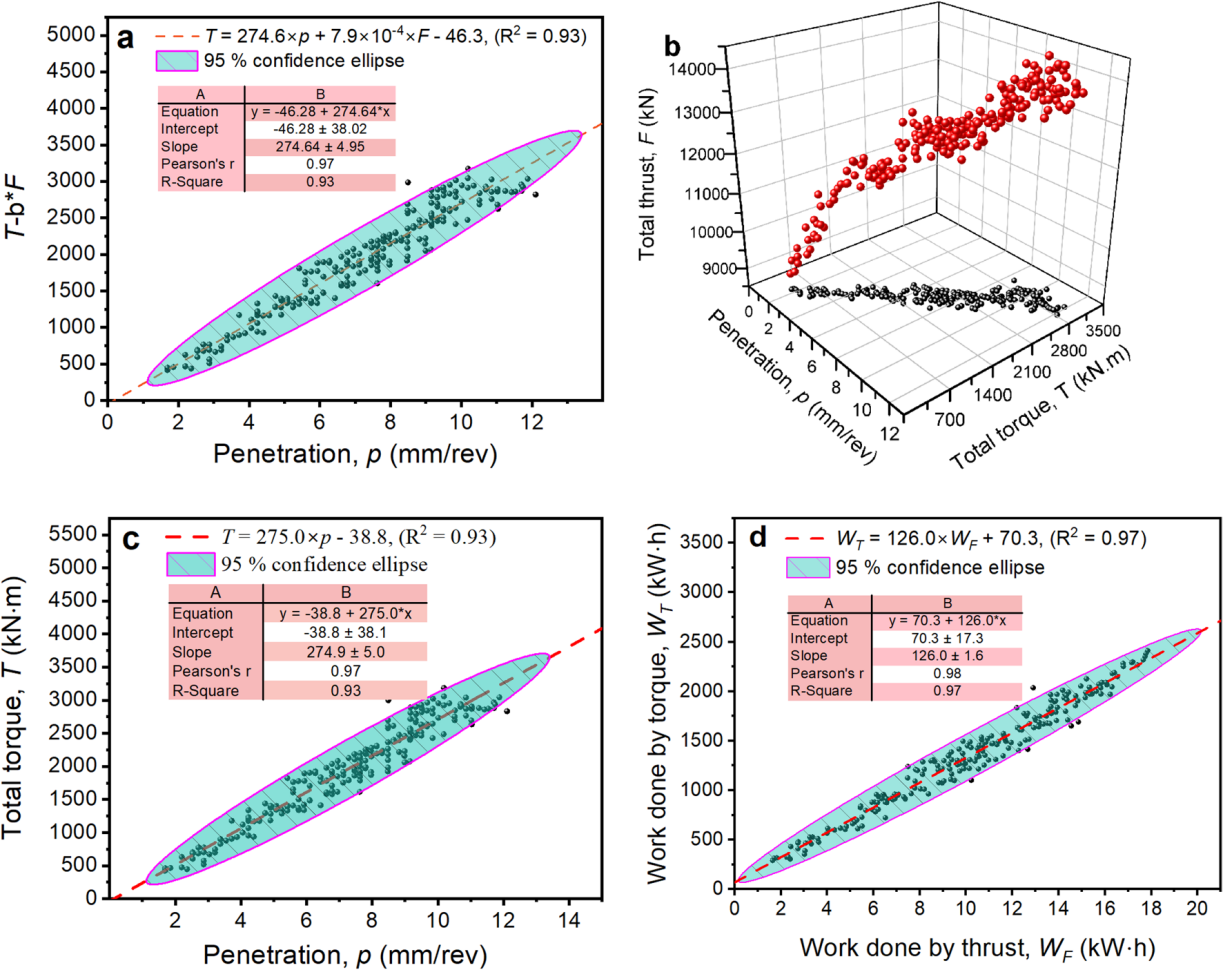
Performance of the three propositions. (**a**) bilinear relation between *T* and (*F*, *p*), (**b**) *T*, *F*, *p* relation in 3D space, (**c**) Linear relation between *T* and *p*, and (**d**) Linear relation between *W*_*T*_ and *W*_*F*_.

The three propositions in “Overview” section are examined using the data of a tunnelling cycle with Chainage No. 66912. The physical and mechanical indexes of the rock mass during this cycle are presented in Table 5. The rock was granite, and the surrounding rock class was III. Figure 8a–d show the performance of the three propositions. The results are as follows.

1. Fig. 8a shows the fitting effect of (*T*-b*F) and *p* in this tunnelling cycle. The goodness of fit (*R*^2^) was 0.93. The Pearson correlation coefficient (r) was 0.97. The root mean square error (RMSE) was 182.32, which is small compared with the Y values of the data points, and the 95% confidence ellipse of the fitting line covers most of the measured data points. These results suggest that the fitting line can well reflect the relationship between (*T*-b*F) and *p*, with an intercept of − 46.3, and the standard error was 38.02, that is, the intercept was in the range (− 84.3, 8.26), which is slightly negligible compared to the Y values. Figure 8b shows the 3D relationship between *T*, *p*, and *F*. Thus, Fig. 8a,b prove that Proposition 1 is true for Chainage No. 66912.
2. As shown in Fig. 8c, *T* was positively correlated with *p*. The fitting results of *T* and *p* show that the r was 0.97, which implies that *T* is strongly correlated with *p*. The *R*^2^ was 0.93, and the mean absolute percentage error (MAPE) was 8.1%, which implies that the current linear fit formula was reasonable. Moreover, the intercept was in the range (− 76.84, 0.72), which is negligible compared to the Y value. Therefore, within the current tunnelling cycle the relationship between *T* and *p* conforms to the description of Proposition 2.
3. As shown in Fig. 8d, the work done by torque (*W*_T_) and the work done by torque (*W*_F_) obey a linear relationship relatively well. Furthermore, according to the fitting results, the intercept takes a range of (52.98, 87.64), which is negligible relative to the Y value. Therefore, we can prove that in this tunnelling cycle, *W*_T_ and *W*_F_ conform to a linear relationship and pass through the origin.

**Table 5:** Physical and mechanical properties of rock masses at Chainage No. 66912.

Natural density	Elastic modulus	Poisson’s ratio	Compressive strength	Tensile strength	Shear strength		Quartz content
					Cohesion	Inter nal friction angle	
2.75 g/cm^3^	50.8 GPa	0.24	125.75 MPa	5.29 MPa	4.69 MPa	55.48	15.0%

It must be acknowledged that validating a single tunnelling cycle makes little sense. Only relationships developed in most tunnelling cycles have meaning and are valuable. If one relationship holds in most tunnelling cycles, it can be regarded as universal and may be an objective law.

#### Verification by the data reported in Gong et al.^48^

Although the proposed models performed well in the TBM3 LOT of the YSP, they should be investigated in other projects. The rock in the YSP is mostly in classes II to IV, and the tunnel overburden is primarily in 50–100 m and no more than 260 m. The performance of these models may be altered if RCs vary in other projects.

Gong et al.^48^ reported detailed boring data of a TBM used in a deep tunnel sewerage system (DTSS) project in Singapore. Unfortunately, only data from one tunnelling cycle was available in this study. The data were used to verify the proposed model.

**Figure 9:**
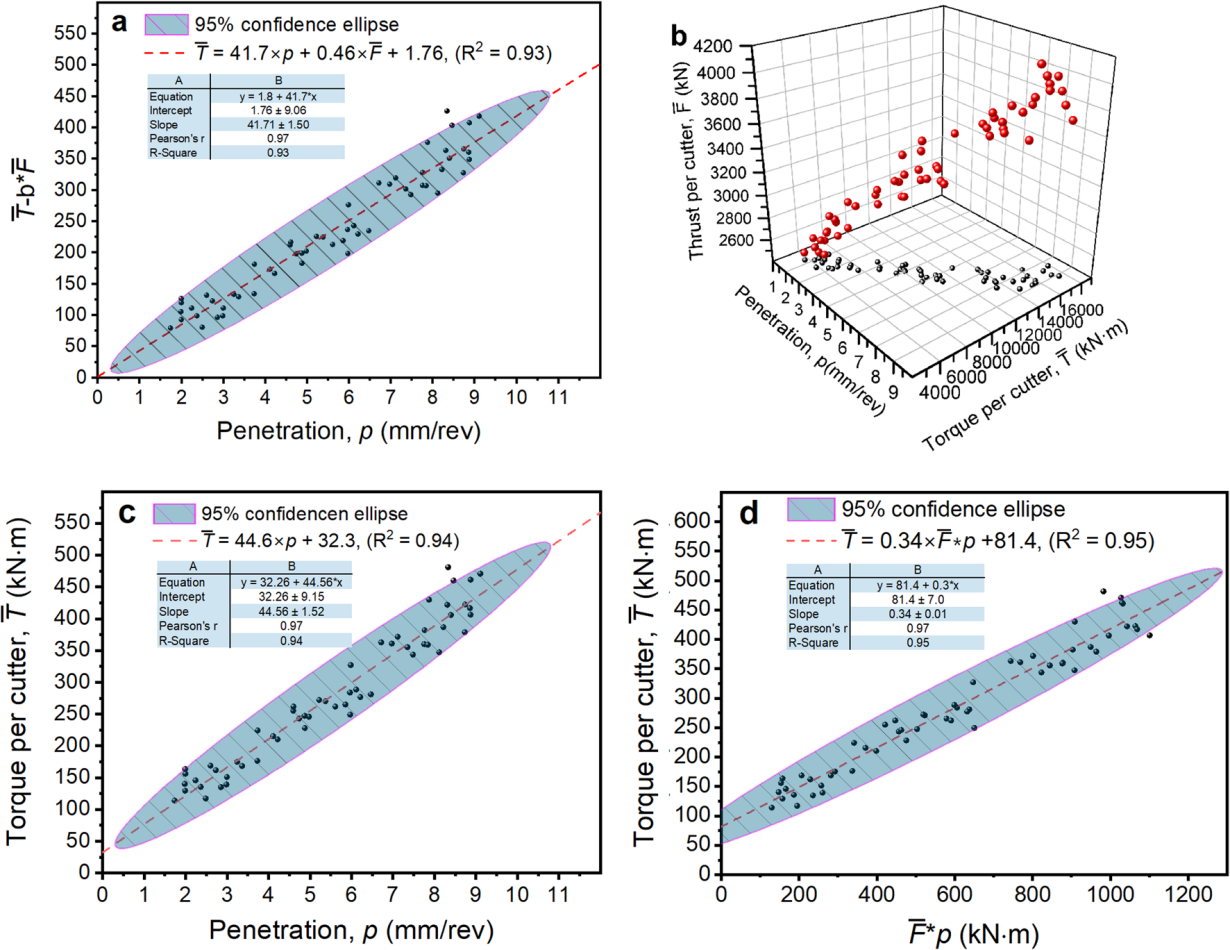
Performance of the proposed model in DTSS project reported by Gong et al.^48^. (**a**) bilinear regression results (Proposition 1), (**b**) *T*-*F-p* relation in 3D space, (**c**) *T*-*p* relation (Proposition 2), and (**d**) *T*-F*p relation (Proposition 3).

The total number of disc cutters in DTSS was 33. The average torque and thrust of the disc cutter, that is, the values of $T/N$ and $F/N$, were recorded in detail. These data were used to verify the performance of the two propositions, and the results are shown in Fig. 9. The results demonstrate the following.

i. As shown in Fig. 9a, the bilinear relationship between *T*, *F,* and *p* has a goodness of fit of 0.94. The 3D effect is shown in Fig. 9b. They verified that Proposition 1 is applicable.
ii. As shown in Fig. 9c, a good linear correlation between the torque per cutter and penetration was found, which verifies that Proposition 2 (the *TPI* concept) is applicable.
iii. As shown in Fig. 9d, the torque per cutter has a good linear relationship with the product of the thrust per cutter and penetration. This relationship is equivalent to the expression in Proposition 3.

## Model adaptation test based on TBM construction data in YSP

Data from 12,237 tunnelling cycles in the YSP were used to assess the applicability (or validity) of existing models and the three newly proposed energy consumption relations. Furthermore, better models will be developed and used to establish a TBM RFI system via a performance comparison.

### Metrics of model performance

To compare the performances of the various models, first, model performance metrics were established. The following two variables were used to assess the model’s performance in this study.

i. Goodness of fit, *R*^*2*^. A model is preferable if it has a higher *R*^*2*^ value when fitting data. In this study, the data for 12,237 tunnelling cycles were fitted by the candidate models, and 12,237 *R*^*2*^ values were statistically analysed. $\mathcal{P}1$**,** the proportion of fitting goodness *R*^*2*^ > 0.6, was used to compare the performance of the various models.
ii. Physical meaning of fitting parameters. Typically, the fitting parameters for a rock fragmentation model are expected to have distinct physical meaning and reflect the rock state. Thus, the fitting parameter on a physical basis should be positive. From this point, $\mathcal{P}2$, the positive rate, indicates rationality. Two metrics were utilised to evaluate a model’s performance with the above consideration, as listed in Table 6.

**Table 6:** Indicators used to evaluate the performance of a model.

Symbol	Definition	Formula
$\mathcal{P}1$	Percentage of *R*^*2*^ > 0.6	$\mathcal{P}1$= Count (*R*^*2*^ > 0.6)/NS①
$\mathcal{P}2$	Positive rate	$\mathcal{P}2$= Count (${x}_{i} > 0$)/NS①

*NS* represents the total number of samples (12,237 in this study), “Count (if true)” is the counting function, $x$ is the fitting parameter, $i$ is the number of samples, ${x}_{i}$ is the $x$ value for the $i$ sample.

### Model adaptation test results

#### Relation between cutterhead torque and penetration

**Figure 10:**
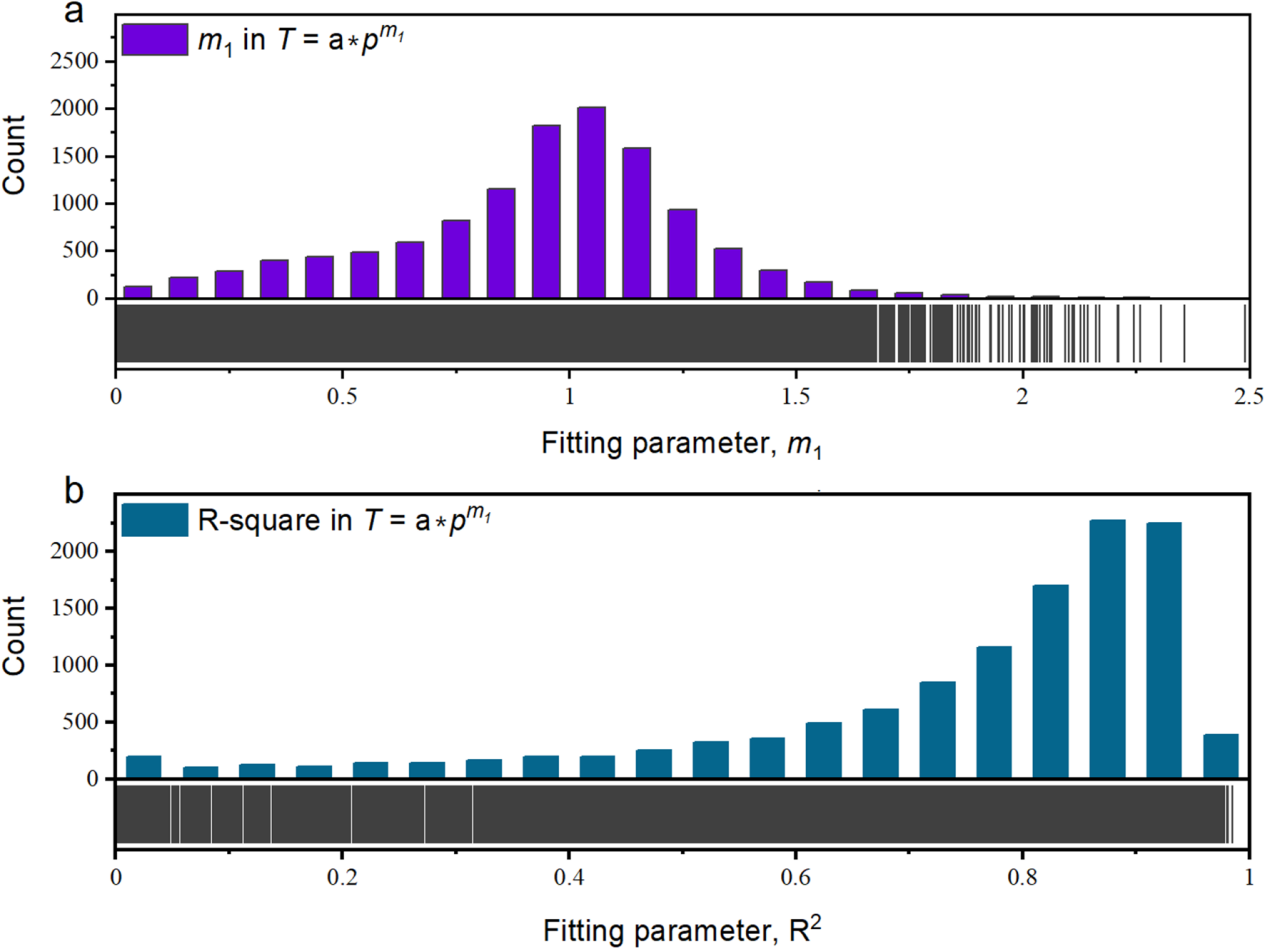
Performance of torque-penetration relation (Eq. 4.1) in TBM3 LOT. (**a**) count of fitting parameter, *m*_1_, and (**b**) count of fitting parameter, R^2^.

First, the relationship between cutterhead torque and penetration was investigated. Equation 4.1 fits all the data from 12,237 tunnelling cycles. For each tunnelling cycle, the *m*_1_ value could be obtained by regression. Subsequently, *m*_1_ values from 12,237 cycles were statistically analysed, and the results are shown in Fig. 10a. It can be observed that *m*_1_ has a mean of 0.98 and standard deviation of 0.47. As shown in Fig. 10b, the goodness of fit data for these 12,237 tunnelling cycles was also statistically assessed. Fitting goodness *R*^*2*^ values greater than 0.6 are found in 74.1% of tunnelling cycles. That is, this formula is correct for the majority tunnelling cycles in the TBM3 LOT.

Several possible reasons can contribute to an *R*^*2*^ value below 0.6 in a tunnelling cycle, including the data dispersion caused by changing rock properties in the same penetrating advance (interlayers, faults, water, and other factors). As the disc cutter intrudes into the rock, the torque progressively increases with the increase in penetration. In some tunnelling cycles the *m*_1_ values are smaller than 0, this goes against the rock fragmentation mechanism. These data were considered abnormal and should be eliminated. After removing these data, the mean *m*_1_ was 1.01, standard deviation was 0.41, and *m*_1_ values followed a normal distribution. This mean value of *m*_1_ is very close to 1, which is substantially less than 1.5 recommended by^47^ (Appendix 2).

#### Relation between total thrust and penetration

**Figure 11:**
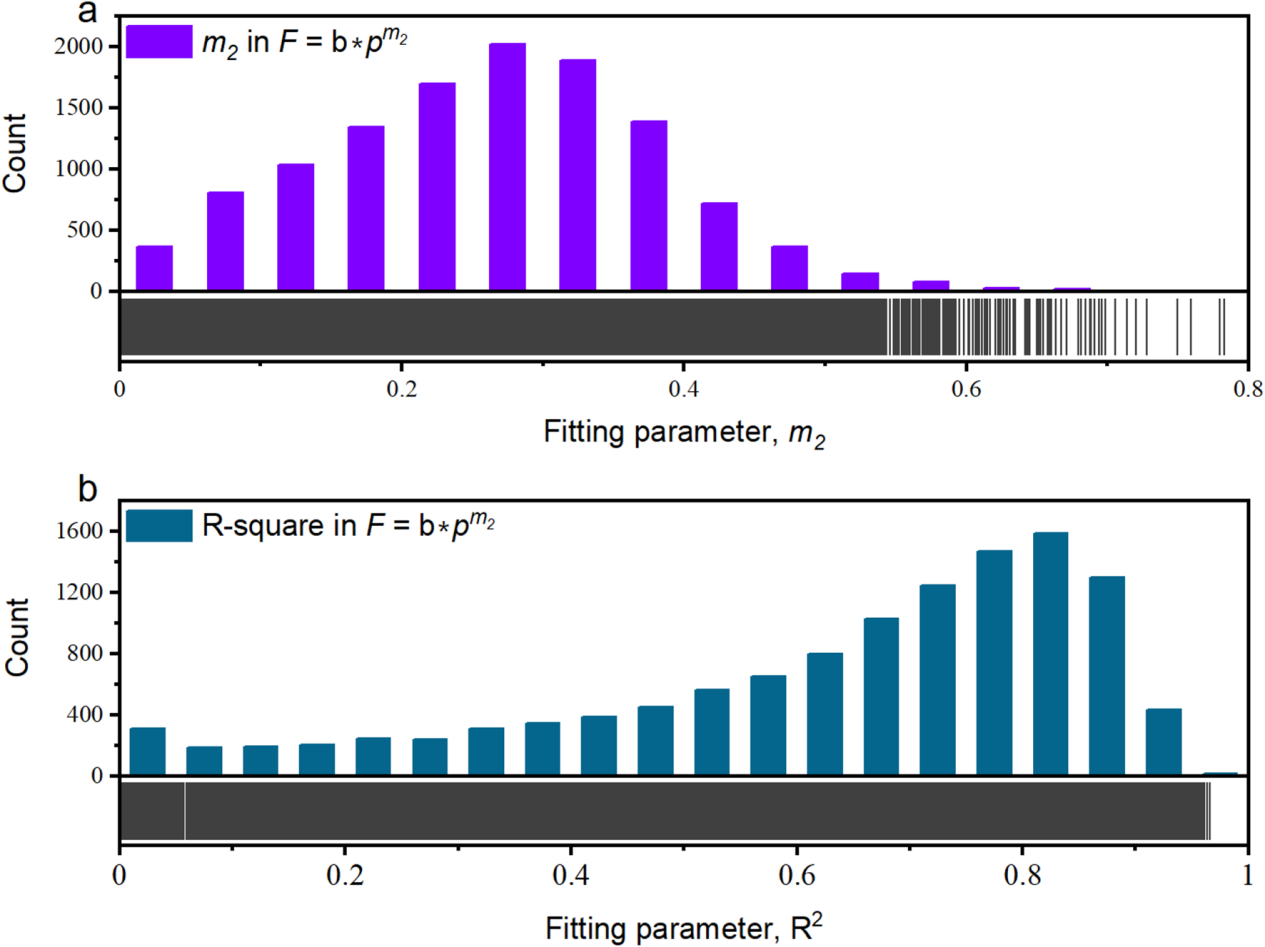
Performance of thrust-penetration relation (Eq. 4.2) in TBM3 LOT. (**a**) count of fitting parameter, *m*_2_, and (**b**) count of fitting parameter, R^2^.

Equation 4.2 is used to evaluate the relationship between the total thrust and penetration, as in the case of the torque. The results are shown in Fig. 11. *R*^*2*^ values greater than 0.6 are seen in approximately 64.6% of the tunnelling cycles. Essentially, the regularity of the *F*-*p* relationship in this project is weaker than that of the *T*-*p* relationship. After removing the abnormal data (*m*_2_ values less than 0), the mean *m*_2_ was 0.26, standard deviation was 0.12, and *m*_2_ values followed a normal distribution. The empirical value of *m*_2_ in the NTNU model (Fig. 3.2 of^27^ varied with RC and was (1/1.13, 1/6.26). These values were consistent with the data shown in Fig. 11a.

The mean value of *m*_2_ obtained in this study seems to be smaller than that obtained in previous models of *F*-*p* relation, such as 1 in Fukui and Okubo^47^, 0.5 in Sanio^24^, and approximately 0.5–1.5 in the CSM model^25^. One possible reason is that the required total thrust for critical penetration is different^49,^^50,^^51^. The critical penetration is defined as the rock mass would not break unless the loading was greater than this value and related to the ultimate strength of the rock mass.

The power relation between *F* and *p* adapted to 64.6% of tunnelling cycles. Essentially, this relationship can be utilised as a useful reference for the TBM boring process. However, it lacks a physical basis and power varies among the tunnelling cycles. Further studies should be conducted to identify the physical meaning of the parameters involved in this relationship.

Based on the results in Fig. 11, the traditional definition of *FPI*, which denotes the ratio of the total thrust to penetration, is not constant during a tunnelling cycle. Because the power relationship is valid in this study, we recommend adopting a new definition of *FPI*:

9 $$FPI=\frac{F}{{p}^{{m}_{2}}},$$

where the statistical characteristics of *m*_2_ vary with the selected tunnelling cycles. Using the goodness of fit (*R*^2^) as a selection criterion, the statistical characteristics of *m*_2_ were resolved and are listed in Table 7. Based on the results in Table 7 and a theoretical *m*_2_ value of 0.5 in Sanio^24^, the *m*_2_ value is recommended as 0.3.

**Table 7:** Statistical characteristics of m_2_ in *F*-*p* relation.

Selecting criterion^a^	Count of tunnelling cycles	Statistical characteristics of *m*_2_	
		Mean value	SD
*R*^2^ > 0.8	3334	0.32	0.09
*R*^2^ > 0.6	7875	0.30	0.10
All	12,181	0.26	0.12

^a^*R*^2^ is the goodness-of-fit of Eq. 4.2.

#### Model adaptation test results of existing models

The average tunnelling boring cycle progress in TBM3 LOT is 1.1 m. Assuming the rock mass properties do not vary much, the model formulas in Appendixs 1 and 2 may be simplified to the form in Table 8, and the data from 12,237 tunnelling boring cycles are utilized to fit these models. Table 8 lists the statistical results of *R*^2^ for these models.

**Table 8:** Model universality test of existing models.

Model		Percentage for *R*^2^ (%)^a^				
Ref*	Formula	[1.0, 0.9)	[0.9, 0.8)	[0.8, 0.7)	[0.7, 0.6)	≤ 0.6
Roxborough et al.^23^	$F=a\sqrt{\left(b{p}^{3}-{p}^{4}\right)}$	0	0.1	0.3	0.7	98.9
	$T=a{p}^{2}$	8.4	21.3	15.9	10.0	44.4
	$T/F=a\sqrt{\left(p/\left(D-p\right)\right)}$	2.3	17.0	18.2	12.6	49.9
Ozdemir et al.^25^	$F=a{p}^{1.5}+b{p}^{0.5}$	11.8	21.8	17.3	12.8	36.3
	$T=a{p}^{2}-b{p}^{3}$	36.2	24.0	13.5	8.2	18.1
Sanio et al.^24^	$F=a{p}^{0.5}$	3.1	4.7	9.5	10	72.7
	$T/F=a{p}^{0.5}$	2.0	16.5	18.5	12.7	50.3
Rostami et al.^52^	$F=a\sqrt{\left(\left(D-p\right)/D\right)}$	0	0	0	0	100
	$T=a\sqrt{\left(p/D\right)}$	1.4	16.5	24.3	19.0	38.8
NTNU^27^	$F=a{p}^{{m}_{2}}$	11.8	23.1	16.9	11.6	36.6
	$T/F=a{p}^{0.5}$	2.0	16.5	18.5	12.7	50.3
Fukui et al.^47^	$F=ap$	0.1	1.0	2.6	4.4	91.9
	$T=a{p}^{1.5}$	23.7	22.7	12.2	7.4	34.0
	$T/F=a{p}^{0.5}$	2.0	16.5	18.5	12.7	50.3
Gong et al.^21^	$F=a{p}^{0.25}$	1.7	16.0	19.7	16.3	46.3
Others^b^	$F=ap$	0.1	1.0	2.6	4.4	91.6
Farrokh et al.^33^	$F=alnp$	0.9	4.1	6.1	7.2	81.7
Jing et al.^30^	$F=ap+b$	5.7	22.1	18.6	12.9	40.7
Goodarzi et al.^53^	$F=alnp+b$	10.3	22.7	17.6	12.5	36.9

^a^*R*^2^ is the goodness of fit for a model in a tunnelling cycle; the percentage of *R*^2^ in an interval is the ratio of the number of tunnelling cycles with *R*^2^ satisfying the condition to the total number of tunnelling cycles (12,237 in TBM3 LOT).

^b^The references include Hassanpour et al.^31,36^, Hamidi et al.^32^, and Farrokh et al.^33^.

Referring to Table 8, the following two models perform well in terms of the relationship between cutterhead torque and penetration.

i. Theoretical torque-penetration relation of Ozdemir’s model^25^. This formula employs two fitting parameters that are thought to be related to the RC. Although the model is built on a V-type cutter disc, it performs well in the TBM3 LOT with constant-cross-section disc cutters. In all tunnelling cycles, the fraction of *R*^2^ > 0.6 is 81.9%.
ii. theoretical torque-penetration power relation of Fukui and Okubo model^47^. It involves only one fitting parameter, and its proportion of *R*^2^ > 0.6 is 66.0%.

The following four models perform well in the thrust-penetration relationship.

(i) Again, Ozdemir’s^25^ theoretical thrust-penetration relation shows good performance, with a proportion of *R*^2^ > 0.6 of over 63.0%. It contains two parameters for the fitting.
(ii) The NTNU model’s empirical thrust-penetration power relationship (2000). It features two fitting parameters, with an *R*^2^ > 0.6 proportion of more than 63.0%.
(iii) Logarithmic linear thrust-penetration relation of Goodarzi et al.^53^. The proportion of *R*^2^ > 0.6 is greater than 63.0%.
(iv) The linear thrust-penetration relation in Jing et al. (2009). The proportion of *R*^2^ > 0.6 is also close to 60.0%.

It should be noted that the above test does not consider the application ranges of the various models. Rostami^52^ compared the classical CSM model (an individual cutter model proposed by^25^ and the NTNU model (an integral representative model) and applied them to three tunnel projects. Their results show that coordinated efforts are required when these models are applied to various types of rocks and TBM. Such coordinated efforts were difficult and were not included in this study. Hence, whether the above conclusions are effective in other projects remains to be studied.

#### Model adaptation test results of three newly proposed energy consumption relations

The data of TBM3 LOT were utilised to test the universality of the three newly proposed propositions. Table 9 lists the results of the statistical analysis. The results in Table 9 show that the three newly proposed propositions all have *R*^2^ > 0.6 over 70.0%. Particularly in Proposition 1, the proportion of *R*^2^ > 0.6 is close to 90.0% in all tunnelling cycles.

**Table 9:** Model Universality Test of three newly proposed Energy consumption relations.

Proposition		Percentage for *R*^2^ (%)^a^				
No	Formula	[1.0, 0.9)	[0.9, 0.8)	[0.8, 0.7)	[0.7, 0.6)	≤ 0.6
1	$T={I}_{c}p+{I}_{f}F$	22.2	36.1	19.1	10.3	12.3
2	$TPI=T/p=const$	21.6	32.7	16.5	9	20.2
3^b^	$WR={c}_{0}Tn/Fv=const$	27.4	33.3	13	6.8	19.8

^a^The definition and calculation method are the same as those used in Table 7.

^b^${c}_{0}$ = 6283 (as shown in Eq. 8.3).

Three models describe the relationships between the torque, thrust, and penetration, which are proposed in Proposition 1 (Eq. 6) (a bilinear relation between torque, thrust, and penetration), the formula from^47^ (listed in Appendix 2, as the ratio of torque to thrust is proportional to the square root of penetration), and the newly proposed Proposition 3 (Eq. 8.3), respectively. In this section, the three models are verified using data from 12,237 tunnelling cycles.

**Figure 12:**
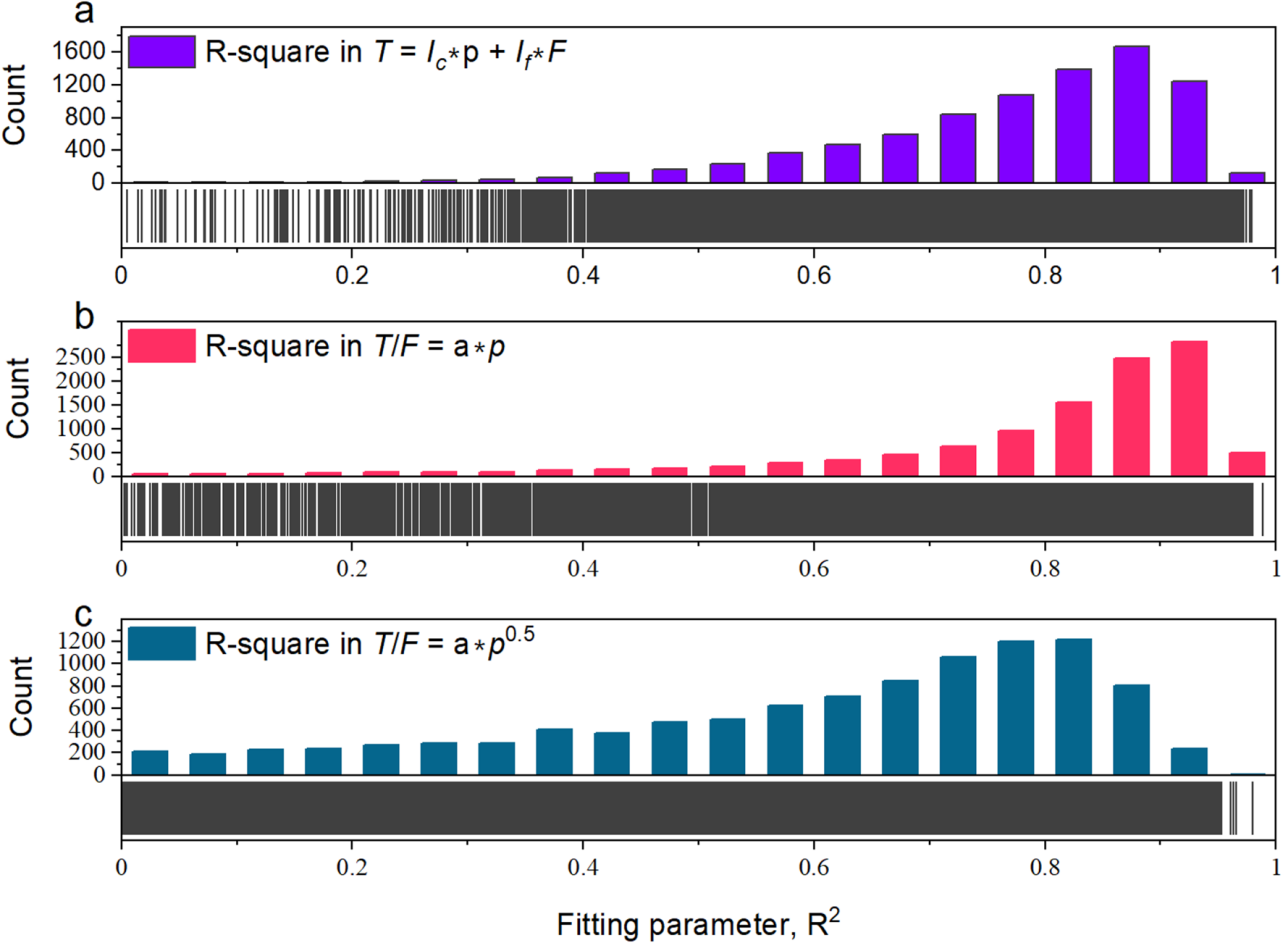
Performance of torque-thrust-penetration relation in TBM3 LOT. (**a**) Proposition 1 (Eq. 6), (**b**) Proposition 3 (Eq. 8.3), and (**c**) Fukui and Okubo’s formula.

The results are shown in Fig. 12. For Proposition 1 (Eq. 6) and Proposition 3 (Eq. 8.3), the percentage of advances with *R*^2^ values larger than 0.6 is all over 80%, which is significantly higher than the formula’s 49.7%^47^. This demonstrates that Proposition 1 (Eq. 6) and Proposition 3 (Eq. 8.3) perform well, and the empirical model proposed by Fukui and Okubo^47^ has a relatively weak application in this project.

### Comparison of the performance of various models

**Figure 13:**
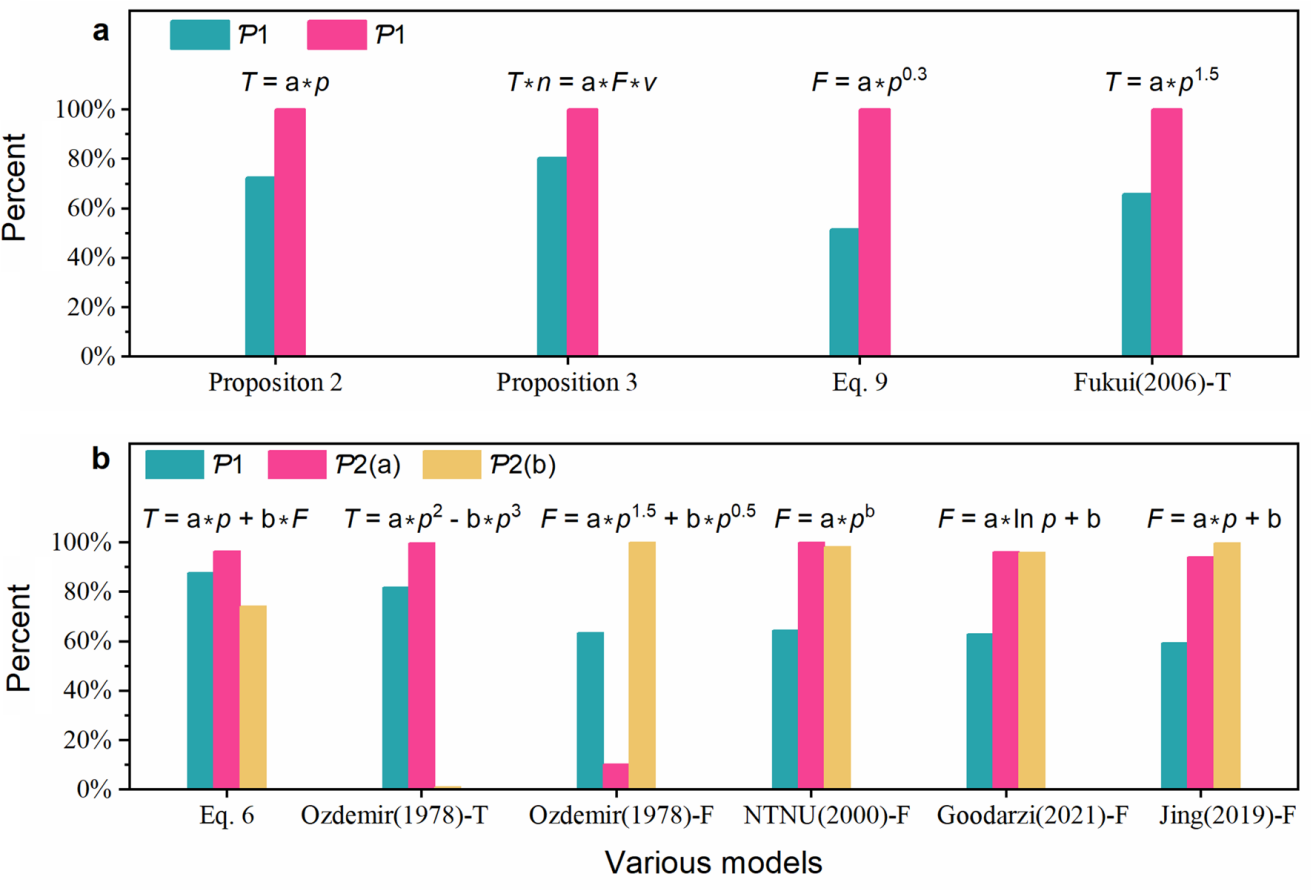
Comparison of statistical results of the various model. (**a**) Single parameter model, and (**b**) double parameter model.

The metrics used to evaluate the performance of the various models, namely $\mathcal{P}1$ and $\mathcal{P}2$ are listed in Table 6. Table 10 lists the results for the models with higher *R*^2^ values (Tables 8 and 9) and compares them in Fig. 13. The findings revealed the following.

(i) Four candidate models are used to characterise the torque-penetration relationship, including Proposition 2 (Eq. 7.4), Fukui^47^–T, Ozdemir^25^–T, and Proposition 1 (Eq. 6). Proposition 2, and Fukui^47^–T are two single-parameter models with higher $\mathcal{P}2$ values. The double-parameter models, Ozdemir^25^–T and Proposition 1 had higher $\mathcal{P}1$ values. Because more parameters are employed, the two double-parameter models fit better and have a higher $\mathcal{P}1$ value. However, their fitting stability was worse than those of the two single-parameter models. Consequently, considering stability, fitting performance, and simplicity, the torque-penetration relationship is advocated in Proposition 2.
(ii) Five candidate models were used to characterise the thrust-penetration relation. The best single-parameter model of the thrust-penetration relation among them still does not have a higher value of $\mathcal{P}1$. Essentially, to describe the thrust–penetration relationship, a double-parameter model should be utilised. However, the performances of the four double-parameter models were quite similar. The Jing^30^–F relation is recommended to characterise the thrust-penetration relation by comparing the fitting stability and simplicity.
(iii) Two candidate models were considered to describe the torque-thrust-penetration relationship. One example is Proposition 3 (Eq. 8.3), with large $\mathcal{P}1$ and $\mathcal{P}2$ values. Proposition 1 (Eq. 6) is the other and has a lower $\mathcal{P}2$ value. Consequently, Proposition 3 (Eq. 8.3) is recommended to characterise the torque-thrust-penetration relationship.

**Table 10:** Evaluation indexes of different models.

	Ref	Formula	Percentage of R^2^ > 0.6	Fitting parameter $\mathrm{a}$	Fitting parameter $\mathrm{b}$
			$\mathcal{P}1$(%)	$\mathcal{P}2$(%)	$\mathcal{P}2$(%)
Torque-penetration model	Equation 7.4	$T=ap$	72.4	100	–
	Fukui^47^–T	$T=a{p}^{1.5}$	65.9	100	–
	Ozdemir^25^–T	$T=a{p}^{2}-b{p}^{3}$	81.9	99.9	1.1
Thrust-penetration model	Equation 9	$F=\mathrm{a}{p}^{0.3}$	51.4	100	
	Ozdemir^25^–F	$F=a{p}^{1.5}+b{p}^{0.5}$	63.6	10.4	100
	NTNU^27^–F	$F=a{p}^{b}$	64.6	100	98.2
	Goodarzi^53^–F	$F=alnp+b$	63.0	96.2	95.9
	Jing^30^–F	$F=ap+b$	59.4	94.1	99.8
Torque-Thrust-penetration model	Equation 6	$T=ap+bF$	87.7	96.4	74.1
	Equation 8.3	$Tn=aFv$	80.4	100	–

Some models have two relationships, one for torque and the other for thrust. Hence, “–T” denotes the torque relationship in this table, while “–F” denotes the thrust relationship. For example, Fukui^47^–T refers to the torque relationship in Fukui^47^ model (see Table 7), whereas Ozdemir^25^–F refers to the thrust relationship.

It should be noted that the comparison in Table 10 shared the same limitation in “Model adaptation test results of existing models” section: no coordinated efforts to various types of rocks and TBM are performed for existing models, and the findings valid in this project are still to be verified in other projects.

### A new TBM rock fragmentation index system

Based on the above results, three models are recommended for characterising the torque-penetration, thrust-penetration, and torque-thrust-penetration relationships. The parameters involved in these three models can be regarded as the RFIs. These parameters are summarised in Table 11, assigned a name, and their physical meaning is described.

**Table 11:** A new TBM rock fragmentation index system.

Name	Formula	Ref	Description
*TPI*	$TPI=T/p$	Proposition 2 (Eq. 7.4)	Torque penetration index
*WR*	$WR={c}_{0}Tn/Fv$^a^	Proposition 3 **(**Eq. 8.3**)**	Work ratio
*AF*	$F=AF*p+BF$	Jing et al.^30^ (Appendix 2)	Thrust to accelerate rock fragmentation
*BF*			Critical thrust for rock fragmentation

^a^${c}_{0}$ equals to 6283 (as shown in Eq. 8.3).

## Benefits of the newly proposed TBM rock fragmentation index system

In “Benefits of the newly proposed TBM rock fragmentation index system” section, a new TBM RFI system is presented. This can be calculated easily using real-time TBM construction data. There is a link between the TBM construction data and RCs. Such a TBM RFI system can be used to overcome the challenge of real-time surrounding rock perception in the narrow-closed workspace of TBM.

This section presents a preliminary study of the relationship between TBM RFIs and rock properties. Owing to the limited space, these preliminary studies did not provide a careful explanation and rigorous demonstration process; instead, they were only used to demonstrate that these indexes can reflect RC to a great extent.

### Relation between TBM rock fragmentation indexes and rock properties

#### Relation between TBM rock fragmentation indexes and collapse probability

**Figure 14:**
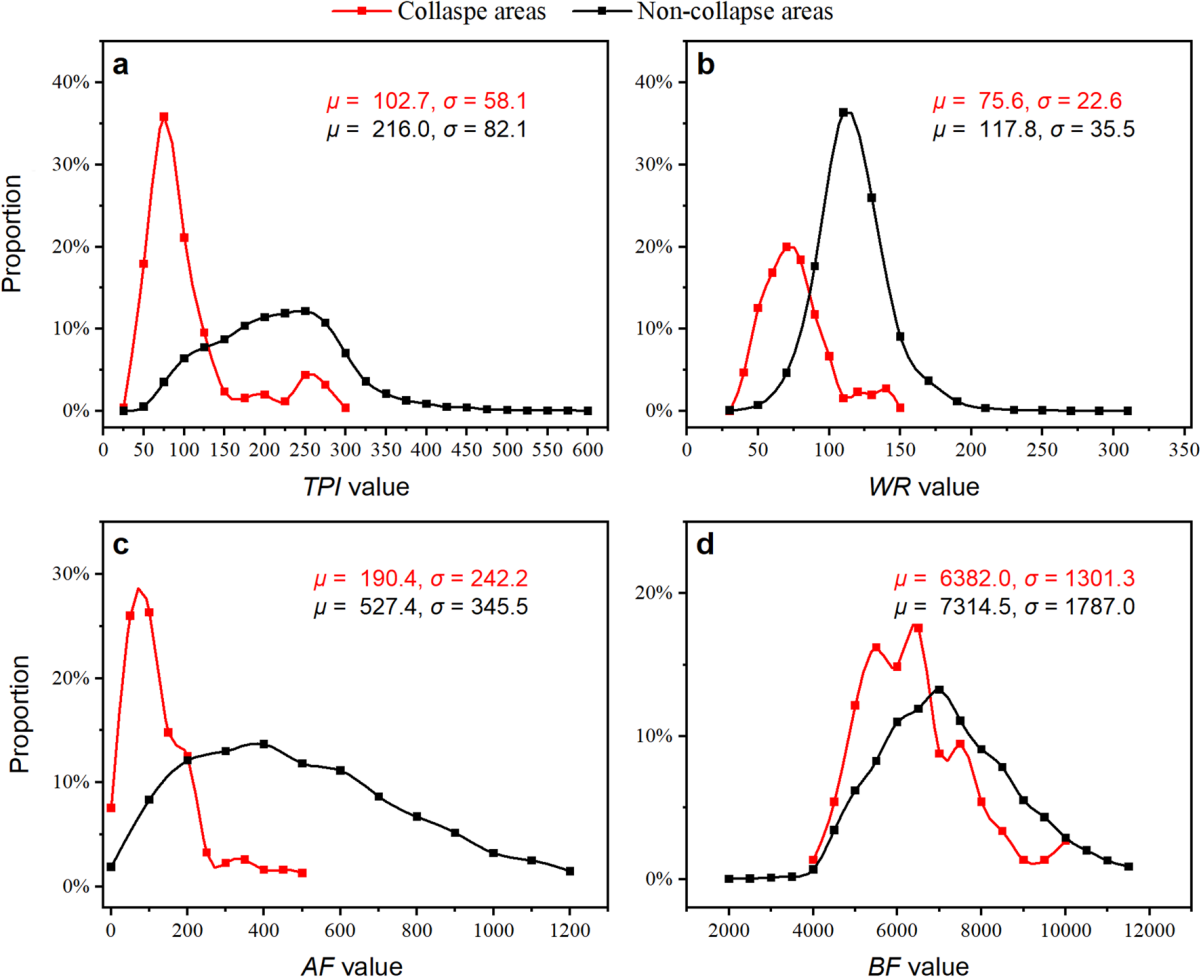
Statistical results of TBM rock fragmentation indexes within and outside collapse zone. (**a**) *TPI*, (**b**) *WR*, (**c**) *AF*, and (**d**) *BF*.

Some collapsed zones were recorded in TBM3 LOT. The rocks in these zones are fractured and unstable. Chen et al.^54^ provided a comprehensive introduction to the collapse zone of YSP. The TBM RFI was statistically analysed both within and outside of these collapse zones, and the results are presented in Fig. 14. *TPI*, *WR*, and *AF* exhibited distinct characteristics within and outside the collapse zones.

#### Relation between TBM rock fragmentation indexes and rock class

The rock class was roughly identified along the entire line of the TBM3 LOT. The TBM RFIs for different rock classes were investigated. A TBM RFI is better if its statistical characteristics vary among rock classes. The TBM RFI is superior if it has a lower coefficient of variation within the same rock class.

**Figure 15:**
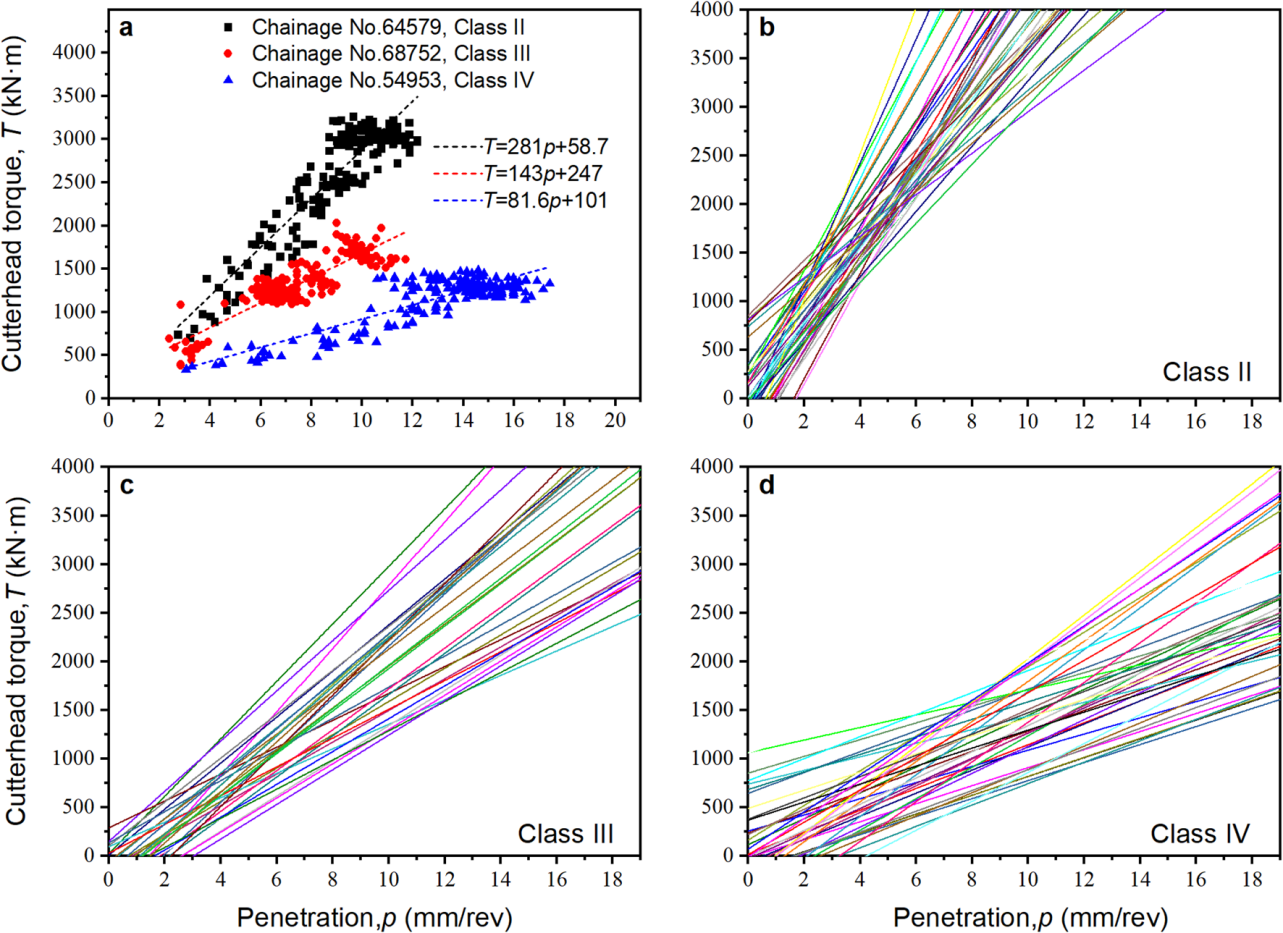
Best-fitted $T$-$p$ relation in various tunnelling cycles. (**a**) Three representative tunnelling cycles, (**b**) Cycles with class II rocks, (**c**) Cycles with rock class III, and (**d**) Cycles with rock class IV.

Consider *TPI* to demonstrate the method of assessing the link between a fitting parameter and the rock class. Figure 15 shows the relationship between *TPI* and rock class as an example. The least-squares method, as shown in Fig. 15a, can be used to generate a best-fit curve for the relationship between torque $T$ and penetration $p$ for each tunnelling cycle. The *T*-*p* best-fitted curves of various tunnelling cycles composed of rock classes II, III, and IV are presented in Fig. 15b–d, where each line represents a best-fitted *T*-*p* relation for a tunnelling cycle. In a tunnelling cycle with a higher rock class, *TPI* or the slope of the *T*-*p* relationship is shown to be lower.

However, the *TPI* values overlap regions for the adjacent rock classes. There are two possible explanations: (i) the rock classification is just crude human judgment, and (ii) *TPI* has a different physical meaning than the rock class. For example, the uniaxial compressive strength of the adjacent rock classes has overlapping values. *TPI* was the same as the uniaxial compressive strength of the rock. Although it is not the only influencing factor, it represents the quality of the rock. More studies are necessary when the new index system is applied to rock categorisation.

**Figure 16:**
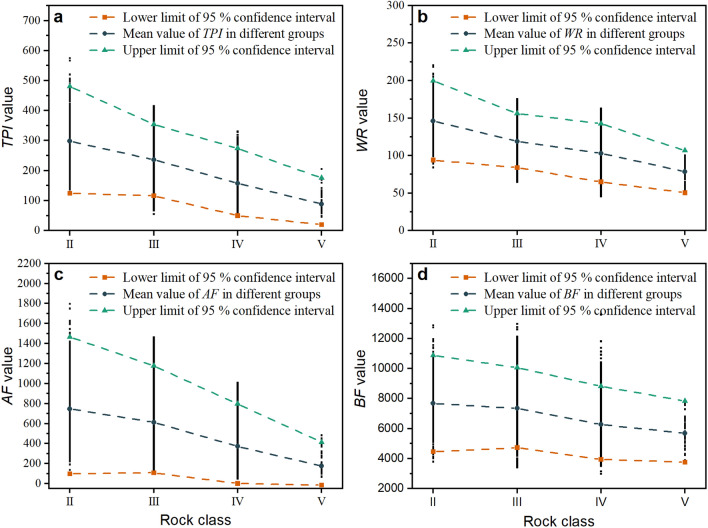
Statistical analysis of TBM rock fragmentation indexes in different rock classes. (**a**) *TPI*, (**b**) *WR*, (**c**) *AF*, and (**d**) *BF*.

By combining all the data of (rock class, *TPI*), as shown in Fig. 16a, a best-fit curve for their relationship and 95.0% confidence ranges can be obtained. As shown in Fig. 16a, the *TPI* values decreased with an increase in rock class. Essentially, the rock is more easily fractured (it has a higher rock class) and its *TPI* values are low. *TPI* can reflect the rock fracture condition to a certain extent. The results (Fig. 16b–c) for *WR* and *AF* decreased as the rock class increased. Essentially, higher-quality rock masses had higher *TPI*, *WR*, and *AF* values. The relationship between *BF* and rock classes was not obvious (Fig. 16d).

### A summary of TBM rock fragmentation indexes

Based on the results of this study, a new TBM RFI system can be proposed as follows

$$TPI,WR,AF,BF.$$

The entire TBM RFI system can reflect the RCs. Each index can be used as an observation angle for RCs. If these indexes vary, then the RCs are considered to change.

*TPI* and *WR* are two indexes that come from a single-parameter model and have a very low fitting error. Therefore, these indexes are more stable and applicable.

*AF* and *BF* originate from the double-parameter model. Their values have certain negative correlations, which weaken the numerical stability of the fitting procedure. If their values are negative, extremely low, or high, these indexes will lose their physical meaning and will be unable to reflect the condition of the rock. Throughout the fitting process, caution should be taken to prevent negative, extremely low, or excessively high values.

*TPI*, *WR*, *AF*, and *BF* show obvious variations under different RCs (including different rock classes, within or outside the collapse zone, and rock with various shear strengths). These four indexes represent the three relationships between *T*-*p*, *F*-*p*, and *T*-*F*-*p*. However, there are some differences between them and the properties of the surrounding rock. *WR* had the highest association, followed by *TPI* and *AF*, whereas *BF* had the weakest association. The index system is composed of four components, and their combination may better reflect the surrounding rock qualities than their usage alone.

### Benefit of the new TBM rock fragmentation indexes

These indexes can be determined immediately during the TBM construction process, without the requirement for additional field or laboratory tests. Thus, the TBM can automatically generate such indexes. These indexes can reflect RCs. Thus, such real-time TBM RFIs can perceive the surrounding rock mass and assist in overcoming the challenge of surrounding rock perception.

RCs can be judged in real time using these TBM RFIs. Furthermore, the control parameter, surrounding rock support measures, and advanced geological detection means can be selected with a solid base. Such TBM RFIs can help boost TBM construction from an empirical and vague stage to a more precise and scientific stage.

It is well known that feature engineering is significantly important for machine learning (or deep learning). We believe that these indexes are suitable candidates for the input parameters in machine learning of TBM data^55^, the prediction of penetration rate^56^, or rock classification^32,^^31,^^37^.

## Conclusions

To overcome the challenge that the perception of RCs in TBM lacks time, space, and methodology, an RFI system was proposed that can be extracted from the real-time monitored rock fragmentation data of the TBM and reflect the RC at the tunnelling face. The major findings of this study are as follows.

This study reviewed existing rock fragmentation models comprehensively and proposed three new rock fragmentation models. Furthermore, the TBM boring data recorded in the YSP were used to assess these models properly. These theoretical and data analyses led to the following conclusions.

1. A comprehensive review and data validation of the relationship between rock fragmentation forces and penetration were conducted in this study. The results demonstrate that, (i) the optimal *T*-*p* model is *TPI*, which is the ratio of torque to penetration, and is a constant in a tunnelling cycle. This relationship is valid for 72.4% of the tunnelling cycles in the YSP. (ii) The optimal *F*-*p* model is one in which thrust is linear to penetration and has a large intercept. *BF* denotes the intercept and *AF* is the slope. This relation is valid for 59.4% of the tunnelling cycles in the YSP. (iii) The optimal *T*-*F*-*p* model is *WR*, which is the ratio between the work done by the torque and the work done by the thrust, and is a constant in a tunnelling cycle. This relation is valid for 80.4% of the tunnelling cycles in the YSP.
2. The parameters involved in these force-penetration relations can reflect the field RCs and can be used as TBM RFIs. A recommended TBM RFI system is proposed, including *TPI*, *AF*, *BF*, and *WR*. Higher *TPI*, *AF*, and *WR* values imply superior rock quality and lower risk of collapse.

With the newly proposed RFI system, the challenge of perception RCs in real time during TBM construction can be significantly alleviated. This system has great potential for categorising rock classes, estimating the risk of surrounding rock collapse, and selecting control parameters or support measures during TBM construction.

## Supplementary Information

Supplementary Information 1.

Supplementary Information 2.

Supplementary Information 3.

## Data Availability

The datasets used and analyzed during the current study available from the corresponding author on reasonable request.

## Abbreviation of rock mechanics parameters

$\xi$: Coefficient of rock friction
${\sigma }_{t}$: Brazilian indirect tensile strength of rock, in MPa
$UCS$: Uniaxial compressive strength of the rock mass, in MPa
*E*: Energy required for rock fragmentation per unit volume, in kJ
${J}_{c}$: Joint condition rating in Rock Mass Rating (RMR) classification system
$\tau$: Unconfined shear strength, in MPa
${I}_{c}$: Rock cohesion index, in kJꞏm^2^
${I}_{f}$: Rock friction index, in m
RC: Rock condition

## Abbreviation of geometric parameters of tunnel boring machine

*A*: Area of the tunnel face, in m^2^
*D*: Diameter of cutter, in m
$\beta$: Contact angle between the rock and cutter, in °
$S$: Spacing between adjacent cutters, in m
${r}_{k}$: Radial distance between center of kth disc cutter and center of cutter head, in m
$r$: Radius of cutter head, in m
$\theta$: Cutter edge angle, in °
$N$: Total number of disc cutter
$\delta$: Cutter tip width, in m

## Abbreviation of mechanical parameters of tunnel boring machine

${f}_{r}$: Rolling force acting on the disc-cutter, in kN
${f}_{n}$: Normal force acting on the disc-cutter, in kN
$\overline{{f }_{n}}$: Average cutter thrust, in kN
$\overline{{f }_{1}}$: Critical thrust to achieve a penetration of 1.0 mm/rev, in kN
$\eta$: Energy transfer ratio of TBM
*TPI*: Torque penetration index, in kNꞏm/(mm/r)
*AF*: Thrust to accelerate rock fragmentation, in kN/(mm/r)
*BF*: Critical thrust for rock fragmentation, in kN
${k}_{c}$: Ratio between the drag force and the thrust force on the cutter
$p$: Penetration, in mm/r
*F*: Total thrust, in kN
$v$: Penetration rate, in mm/min
*T*: Cutterhead torque, in kNꞏm
$n$: Cutter head rotation speed, in r/min
*FPI*: Thrust penetration index, in kN/(mm/r)
*WR*: Work ratio
$\xi$: Coefficient of rock friction
