# Supplementary material for: Rock fragmentation indexes reflecting rock mass quality based on real-time data of TBM tunnelling
Source: Sci Rep. 2023 Jun 27;13:10420. doi: 10.1038/s41598-023-37306-7 (PMC10300193; doi:10.1038/s41598-023-37306-7)
Supplement: Supplementary file 1 — Supplementary Information 1. [file 41598_2023_37306_MOESM1_ESM.docx]

# Appendix 3 Field measured rock physical and mechanical properties

Geologists have conducted many field and laboratory tests on the physical and mechanical properties of rocks, and the results are shown in Table A3.

Table A3 Field-measured values of rock physical and mechanical indices

| Chainage No. | Lithology | $\rho$ | $\omega$ | *UCS* | $k$ | $\sigma_{t}$ | $E_{50}$ | $\mu$ | $E_{0}$ | $p$ | $\tau$ | |
| --- | --- | --- | --- | --- | --- | --- | --- | --- | --- | --- | --- | --- |
|  |  | g/cm^3^ | % | MPa | - | MPa | GPa | - | GPa | % | MPa | ° |
| [48+900, 50+179] | Quartz  Diorite | 2.61 | 0.80 | 78.1 | 0.64 |  |  |  |  |  |  |  |
| [46+700, 50+179] | Quartz  Diorite | 2.72 | 0.23 | 135.1 | 0.74 | 8.0 | 7.0 | 0.21 | 5.5 | 22.0 | 8.0 | 52 |
| [50+179, 56+200] | Granite | 2.68 | 0.72 | 142.8 | 0.63 | 6.0 | 2.9 | 0.22 | 2.0 | 34.0 | 8.0 | 50 |
| [58+970, 60+220] | Tuffaceous  sandstone | 2.62 | 0.46 | 96.8 | 0.62 | 2.5 | 2.0 | 0.30 | 1.0 |  | 5.0 | 45 |
| [60+220, 62+374] | Tuff | 2.70 | 0.14 | 90.9 | 0.66 | 4.0 | 6.3 | 0.29 |  |  | 6.0 | 50 |
| [62+374, 63+884] | Diorite | 2.72 | 0.23 | 120.5 | 0.83 | 5.0 | 5.0 | 0.24 | 3.5 |  | 4.5 | 50 |
| [63+884, 71+046] | Limestone | 2.71 | 0.29 | 106.6 | 0.75 | 3.0 | 3.0 | 0.23 | 3.0 | 15 | 4.0 | 45 |
| [65+978, 67+913] | Limestone | 2.68 | 0.32 | 85.7 | 0.70 |  |  |  |  | 16 |  |  |
| [71+466, 71+855] | Albite  Porphyry | 2.62 | 0.26 | 90.0 | 0.78 |  |  |  |  |  |  |  |
| [71+466,71+855] | Glutenite | 2.62 |  | 65.0 |  |  |  |  |  |  |  |  |

Note: $\rho$, $\omega$, *UCS*, $k$, $\sigma_{t}$, $E_{50}$, $\mu$, $E_{0}$, $p$, and $\tau$ denote natural density, water absorption, uniaxial compressive strength, softening index, tensile strength, deformation modulus, Poisson’s ratio, elastic modulus, quartz content, and shear strength of the rock mass, respectively.
