# Supplementary material for: Rock fragmentation indexes reflecting rock mass quality based on real-time data of TBM tunnelling
Source: Sci Rep. 2023 Jun 27;13:10420. doi: 10.1038/s41598-023-37306-7 (PMC10300193; doi:10.1038/s41598-023-37306-7)
Supplement: Supplementary file 2 — Supplementary Information 2. [file 41598_2023_37306_MOESM2_ESM.docx]

# Appendix 1 Individual cutter models

Appendix 1 Rock fragmentation models for forces acting on a single cutter disc

| Cuter | Reference | Formula |
| --- | --- | --- |
| V-type | Roxborough and Phillips (1975) | $f_{n}=4\sigma_{c}\tan\theta/2\sqrt{2rp^{3}{-p}^{4}}$, $f_{r}=4\sigma_{c}p^{2}\tan\theta/2$, $CC=f_{r/f_{n}}=\tan\Phi/2=\sqrt{p/\left( D-p \right)}$ |
|  | Ozdemir and Miller (1978,  the famous CSM model) | $f_{n}=D^{0.5}p^{1.5}\left[ 4/3 \sigma_{c}+2\tau\left( S/p-2\tan\theta/2 \right) \right]\tan\theta/2$,$CC=f_{r/f_{n}}=\tan\beta\approx\tan\Phi/2=\sqrt{p/\left( D-p \right)}$  $f_{r}=\left[ \sigma_{c}p^{2}+\left( 4\tau\Phi p^{2}\left( S-2p\tan\theta/2 \right) \right)/D\left( \Phi-\sin\Phi\cos\Phi\right) \right]\tan\theta/2,$ |
|  | Sanio (1985) | $f_{n}=Sk\tan\theta/2\sqrt{DSp}$, $CC=f_{r}/f_{n}=4/5\left( p/D \right)^{0.5}$ |
| CCS | Rostami and Ozdemir (1997) | $f_{t}=2.12\delta r\Phi\sqrt[3]{\left( {\sigma_{c}}^{2}\sigma_{t}S \right)/\left( \Phi\sqrt{\delta r} \right)}$, $f_{n}=f_{t}\cos\Phi/2$, $f_{r}=f_{t}\sin\Phi/2$, $CC=\frac{f_{r}}{f_{n}}=\tan\beta\approx\tan\Phi/2=\sqrt{p/\left( D-p \right)}$ |
|  | Gong et al. (2007, 2009) | $f_{n}/p=BI$, $BI\approx{BI}_{\left( 1 \right)}p^{-0.75}$ |
|  | Hassanpour et al. (2009, 2010) | $f_{n}/p=FPI$, $FPI=exp \left( 0.004UCS+0.008RQD+2.077 \right)$ |
|  | Hademi et al. (2010) | $f_{n}/p=FPI$, $FPI=4.161+0.091UCS+0.077RQD+0.117J_{c}+1.077log\alpha\left( R^{2} = 0 . 87 \right)$ |
|  | Farrokh et al. (2012) | $p=\exp\left( 0.41+0.404D-0.027D^{2}+0.0691{RT}_{c}-0.00431UCS+0.0902{RQD}_{c}+0.000893f_{n} \right) \left( R^{2}=0.63 \right)$ |
|  | Goodarzi et al. (2021) | $f_{n}=106.72ln p-100.4$ $\left( R^{2}=0.76 \right)$ |

Note: (i) Rock property parameters: $\sigma_{c}$ and *UCS* represent uniaxial compressive rock strength, $\sigma_{t}$ represents Brazilian indirect tensile strength of rock, $\tau$ represents unconfined shear strength, $J_{c}$ is the joint condition rating in Rock Mass Rating system, *RQD* denotes rock quality designation.

(ii) Cutter geometry parameters: *θ* is the cutter edge angle, *r* is the cutter radius, *D* is the diameter of the cutter, *δ* represents the cutter tip width.

(iii) Cutterhead parameters: *S* represents the spacing between the adjacent cutters.

(iv) Rock fragmentation parameter: *Φ* is the contact angle between the rock and cutter, which usually differs from $\theta$; and $\beta$ is the angle between the resultant and normal forces. For simplicity, $\beta$ is equal to $\Phi/2$ in many models, $p$ is the penetration, BI_(1)_ represents the critical thrust to achieve a penetration of 1.0 mm/rev (kN/cutter), and BI and FPI represent the field penetration index, which is the ratio of the cutter thrust to the penetration.
