# Supplementary material for: Rock fragmentation indexes reflecting rock mass quality based on real-time data of TBM tunnelling
Source: Sci Rep. 2023 Jun 27;13:10420. doi: 10.1038/s41598-023-37306-7 (PMC10300193; doi:10.1038/s41598-023-37306-7)
Supplement: Supplementary file 3 — Supplementary Information 3. [file 41598_2023_37306_MOESM3_ESM.docx]

# Appendix 2 Empirical holistic models

Appendix 2 Empirical models for cutterhead

| Reference | Formula |
| --- | --- |
| Bruland (2000, the famous NTNU model) | $p=\left( \frac{\bar{f_{n}}}{\bar{f_{1}}} \right)^{b}$, $T={0.5r}_{k}DN\bar{f_{n}}k_{c}$, $CC=f_{r}/f_{n}=CC^{'}\sqrt{p}$ |
| Fukui and Okubo (2006) | $F=c_{1}\sigma_{c}p$, $T=c_{2}\sigma_{c}p^{1.5}$, $T/F=c_{2}/c_{1}p^{0.5}$ |
| Delisio et al. (2013) | $F/p$=$FPI$, $FPI=5952-1794\ln J_{v}+UCS$ $\left( R^{2}=0.78 \right)$ |
| Jing et al. (2019) | $F/N=ap+c$, $a=0.02J_{v}^{2}-1.22J_{v}+19.83$, $c=1.27UCS-0.02J_{v}^{2}-1.12J_{v}+66.19$ |

Note: $\sigma_{c}$ and *UCS* represent uniaxial compressive rock strength; $J_{V}$ is the rock mass per unit volume (number/m^3^); $\bar{f_{n}}$ represents the average cutter thrust (kN/cutter); $\bar{f_{1}}$ represents the critical thrust to achieve a penetration of 1.0 mm/rev; *k*_c_ is the ratio between the drag force and the thrust force on the cutter; *b* represents the penetration coefficient; *k*, $c_{1}$, and $c_{2}$ are constants; and $r_{k}$ is the distance between the centre of the kth disc cutter and the centre of the cutterhead.
